# A Structural Equation Approach to Characterizing Growth and Nonlinearity Underlying Distortion Product Otoacoustic Emissions

**DOI:** 10.1007/s10162-026-01057-9

**Published:** 2026-06-15

**Authors:** Shawn S. Goodman, M. Ehsan Khalili, Julia H. Roemen, Ishan Bhatt, Skyler G. Jennings, Jeffery T. Lichtenhan, Sumitrajit Dhar

**Affiliations:** 1 Department of Communication Sciences and Disorders, University of Iowa, Iowa City, IA USA; 2 Department of Communication Sciences and Disorders, University of Utah, Salt Lake City, UT USA; 3 Dept. of Otolaryngology, University of South Florida, Tampa, FL USA; 4 Turner Scientific, Jacksonville, IL USA; 5 Roxelyn and Richard Pepper Dept. of Communication Sciences and Disorders, Northwestern University, Evanston, IL USA; 6 Knowles Hearing Center, Northwestern University, Evanston, IL USA

**Keywords:** Structural equation model, Latent growth model, Latent growth curve analysis, Generalized linear regression, Rician, Input–output

## Abstract

**Purpose:**

Distortion product otoacoustic emission (DPOAE) magnitudes measured in the ear canal in response to a range of primary stimulus levels as growth functions (GFs) may be useful for assessing cochlear non-linearity, predicting behavioral audiometric thresholds, estimating loudness perception, and differentiating types of cochlear pathology. A variety of stimulation schemes have been proposed, and GF shapes differ depending on the stimulation scheme used. A clearer understanding of the relationships between stimuli, GFs, cochlear non-linearities, and cochlear health is important for maximizing the diagnostic potential of DPOAEs.

**Methods:**

Latent growth modeling, a technique within the structural equation modeling framework, can provide insight into the relationships between observed (e.g., GFs) and unobserved latent variables (e.g., cochlear non-linearities). We describe a latent growth model for characterizing GFs with a generalized logistic function representing the latent non-linearity, coupled with a generalized linear regression model appropriate for fitting GFs with varying signal-to-noise ratios (SNRs). The model was applied to GF data from twelve young adult ears (9 female, 3 male). The resulting fits inferred the shape of the underlying non-linearity and also quantified standard GF characteristics such as slope, threshold, and inflection points.

**Results:**

Data from participants, along with Monte Carlo simulations, demonstrate that this fitting method performs well under low SNR conditions and accurately predicts DPOAE magnitudes at low stimulus levels.

**Conclusion:**

This report establishes a robust method for characterizing GFs, supporting the long-term goal of applying the method in future studies of the relationships between acoustic stimuli, GFs, cochlear non-linearities, and cochlear health.

## Introduction

Intermodulation distortion products (DPs) occur when waveforms of two or more frequencies are processed simultaneously by a nonlinear system, such as the healthy cochlea. Some of this intracochlear DP energy makes its way from the cochlea back to the ear canal, where it can be measured acoustically as distortion product otoacoustic emissions (DPOAEs). Because the healthy cochlea is highly nonlinear, DPOAEs have been widely used as an indirect, noninvasive assay of cochlear function. Growth functions (GFs; also referred to as a level series or input–output functions) describe the relationship between DPOAE magnitude and sound stimulus level. GFs have been used to infer cochlear nonlinearity, predict behavioral audiometric thresholds and loudness perception, and detect subclinical damage related to aging, noise exposure, and ototoxicity [1–8].

Several schemes have been proposed for selecting GF stimulus levels. The pair of evoking pure-tone stimuli, known as primaries, can be presented so that their amplitudes are kept a fixed-dB difference apart [9, 10], or they can be presented at some non-constant dB difference (e.g., the common “scissors” paradigm; [11, 12]). Alternatively, one primary may be fixed in level while the other varies [13, 14]. Regardless of which stimulation scheme is used, it is assumed that observed DPOAEs are closely related to the unobserved nonlinear vibrations of cochlear structures. However, GF shapes differ depending on the stimulation scheme used, complicating inferences about the underlying cochlear non-linearities and associated cochlear health. A clear and accurate understanding of the relationships between acoustic stimuli, GFs, cochlear non-linearities, and cochlear health is important for maximizing the diagnostic potential of DPOAEs.

Recent technical advances have provided tools to strengthen our understanding of these relationships. Stimuli swept in level produce continuous GFs across a range of stimulus levels [15, 16]. These swept paradigms produce essentially the same results as traditional discrete measurements with potentially denser sampling along the x-axis. Advances in stimulus calibration have made possible more precise acoustic measurements, particularly at high frequencies [17–19]. Recent work with the fixed *L*_1_ stimulus scheme suggests that the resulting GFs may have slopes that differentiate types of cochlear hearing loss [3]. Measurements using optical coherence tomography (OCT) are refining our understanding of the relationship between GFs and cochlear vibration patterns in animal models [20]. In spite of these advances, understanding the relationship between GFs and the underlying cochlear non-linearities that create them remains a challenge, particularly in human ears, where the vibration of cochlear structures cannot be directly observed.

Structural models are a broad class of statistical model which can provide insight into the relationships between observed and unobserved (latent) variables. Such models may be particularly useful when prior information informs the mathematical model of the underlying, unobserved structure. A body of previous work shows that cochlear vibration patterns can be well-characterized by saturating sigmoidal functions [21–27]. Such non-linearities have most often been applied to specific cochlear structures or processes (e.g., hair bundle displacement or receptor potential). DPOAE generation is a spatiotemporally distributed process that includes contributions from increasingly broad cochlear regions as stimulus level increases, with DPOAEs potentially being influenced by wave interference between DPs from different locations [28, 29]. Such complex interactions may preclude a full understanding based on a single underlying non-linearity; nevertheless, use of a saturating non-linearity as the latent structure in a structural model may provide insights linking GFs, the underlying cochlear non-linearities, and cochlear health.

We developed a latent growth model (a technique within the structural equation modeling framework [30]) for characterizing DPOAE GFs. GFs were modeled as a function of stimulus level acting on an underlying nonlinear growth process. The model used a generalized logistic function to represent the latent non-linearity, coupled with a generalized linear regression model incorporating the Rician distribution, which is appropriate for fitting GF magnitudes with varying signal-to-noise ratios (SNRs). The best parameter values for the non-linearity were fitted to GF data from individual ears. The output of the model yielded predicted GFs, which facilitated quantification of commonly considered GF characteristics such as slope, threshold, and inflection points. The main objective of this report was to establish robust methods for characterizing GFs, with the long-term goal of applying the methods in future studies of the relationships between acoustic stimuli, GFs, cochlear non-linearities, and cochlear health.

## Methods

### Participants

DPOAE GFs were measured from the right ears of 12 adult participants (9 female, 3 male; mean = 22.9 yr, SD = 1.23 yr, range = 20–25 yr). Participants reported a negative history of hearing problems and listening difficulties. Participants’ ear canals were free of cerumen accumulation, and eardrums were visually normal by otoscopic inspection. Middle ear function was normal as assessed by 226-Hz tympanometry. Participants passed a DPOAE screening (both primaries swept continuously in frequency, *f*_2_ = 0.75–5 kHz, *f*_2_/*f*_1_ = 1.22, *L*_1_ = 65 FPL, *L*_2_ = 55 dB FPL), with peaks in amplitude fine structure ≥ 10 dB SPL from 1–4 kHz. Audiometric thresholds were not tested, since the study was focused on participants with reasonably strong DPOAE magnitudes, rather than with clinically normal behavioral thresholds, per se. The research protocol was approved by the University of Iowa Institutional Review Board, and written informed consent was obtained from all participants.

### Hardware and Calibration

Stimuli were presented and ear canal responses measured using an ER10X probe system (Etymotic Research). The probe system was connected to a 24-bit sound card (RME Fireface 802). Playback and recording were controlled by custom MATLAB software [31]. The sampling rate was 96 kHz, and recordings were streamed to computer disk for post-hoc analysis. A critical part of obtaining high-quality DPOAE recordings is ensuring probe stability. The ER10X probes were inserted into the canal and then stabilized by attaching the probe cord to a headband (3M H10A Peltor Optime earmuffs with the outer plastic and foam core removed to leave a donut shape surrounding the pinna). Thevenin source calibration [32] was completed for each ER10X probe prior to data collection. In each ear, an *in-situ* calibration routine calculated the characteristic and load impedances. The loudspeaker voltage drive was adjusted to deliver the stimulus at the desired forward pressure level (FPL) at each test frequency. For the longer, modified test paradigm (described below), the *in-situ* calibration routine was repeated every 3.6 min during the recording session, and the output voltage was adjusted to ensure stable stimulus pressures at the eardrum.

### Test Paradigms

DPOAEs were elicited with *L*_1_ fixed at 65 dB FPL while *L*_2_ was swept continuously from −2 to 72 dB FPL [15]. Each tone sweep presentation was 9 s in length; therefore, *L*_2_ varied at a constant rate of 8.22 dB/s. These rate and duration parameters were chosen to minimize analysis edge effects while maximizing analyzed DPOAE SNR. Each test consisted of M= 24 nine-second presentations, for a total length of 216 s. DPOAE GFs were collected for each participant at a single *f*_2_ frequency between 1–4 kHz (mean = 1817.3 Hz; SD = 847.1 Hz; min = 1056.0 Hz; max = 3881.0 Hz). To maximize SNR and to avoid potential GF notches due to two-component interactions, the value of *f*_2_ was chosen from a peak in the DPOAE amplitude fine structure obtained during the hearing screening [33]. Primary tone ratio was fixed at *f*_2_/*f*_1_ = 1.22.

As described in the next section, each GF obtained from the swept stimulus paradigm was fitted with a smooth curve that captured the essential features of peak regions and predicted DPOAE levels below the noise floor. To test the accuracy of these predictions at low levels, the same 12 participants were tested using a second, modified test paradigm. This modified paradigm included focused averaging at *L*_2_ = 5 dB FPL (*L*_1_ = 65 dB FPL), a level which produced DPOAE amplitudes below the noise floor of the swept paradigm. This single “snapshot” at *L*_2_ = 5 dB FPL provided a representative test of low-level prediction accuracy. In the modified paradigm, two fixed *L*_1_ sweeps were presented, followed by 2 min of continuous averaging with *L*_1_ held constant at 65 dB FPL and *L*_2_ fixed at 5 dB FPL. The interleaving process was repeated 13 times for a total of 26 interleaved sweeps and 26 min of averaging time at the low level (~ 39 min of total recording time). After the recording was completed, the swept and steady portions were de-interleaved and analyzed separately. The collection of swept recordings was analyzed as described in detail below. The steady low-level portion was analyzed using the same basic signal processing methods. The long averaging at *L*_2_ = 5 dB FPL reduced the noise floor by approximately 21 dB, yielding DPOAEs with approximately 10 dB SNR in most participants. Probes were not removed from participants’ ears between tests.

### GF Signal Processing

Recordings were analyzed using ordinary least-squares regression applied to a series of partially overlapping time-windowed waveforms. Each analysis frame was 500 ms in length. Seventy-one analysis frames were considered for each sweep, with each frame centered at a temporal location corresponding to a target *L*_2_ FPL value. Target values ranged from 0–70 dB FPL in 1 dB steps (75% temporal overlap between adjacent frames).

Let the variable ${\boldsymbol{a}}$ represent a column vector of recorded ear canal pressures in an analysis frame (${\boldsymbol{a}}$ of length *n* = 48,000 samples), multiplied by a Hann window. The overdetermined system of equations ${\boldsymbol{X}}{\boldsymbol{b}}={\boldsymbol{a}}$ was considered, where

1 $${\boldsymbol{X}}=\left[\begin{array}{c} {X}_{11} \\ {X}_{21}\\ \vdots \\ {X}_{n1}\end{array} \begin{array}{c} {X}_{12 } \\ {X}_{22} \\ \vdots \\ {X}_{n2 }\end{array}\begin{array}{c}{X}_{13}\\ {X}_{23}\\ \vdots \\ {X}_{n3}\end{array}\right], {\boldsymbol{b}}=\left[\begin{array}{c}{b}_{1}\\ {b}_{2}\\ {b}_{3}\end{array}\right] , {\boldsymbol{a}}=\left[\begin{array}{c}\begin{array}{c}{a}_{1}\\ {a}_{2}\\ \vdots \end{array}\\ {a}_{n}\end{array}\right]$$

The first and second columns in ${\boldsymbol{X}}$ contained Hann-windowed sinusoids of frequency 2*f*_1_-*f*_2_ in cosine and -sine phase, respectively. The third column in ${\boldsymbol{X}}$ was a vector of ones, to account for any mean offset from zero. The vector ${\boldsymbol{b}}$ contained the three unknown coefficients corresponding to the three columns of ${\boldsymbol{X}}$. The best-fitting (in the least squares sense) coefficients, $\widehat{{\boldsymbol{b}}}$, were found by minimizing the matrix norm

2 $$\widehat{{\boldsymbol{b}}}=\underset{{\boldsymbol{b}}}{\text{arg min}}{ \Vert {\boldsymbol{a}}-{\boldsymbol{X}}{\boldsymbol{b}}\Vert }^{2}$$

The estimate of DPOAE magnitude and phase in that analysis frame was expressed in complex rectangular form as

3 $${z}={\widehat{b}}_{1}+i{\widehat{b}}_{2} ,$$

where i is the imaginary operator. In this paper, variables written in script font, such as ${z}$, indicate the variable is complex. The fitting procedure was repeated for each of the M= 24 stimulus sweeps in the same analysis frame, and the complex coefficients were stored in a vector ${\boldsymbol{z}}$ of size 24 × 1 (M= 26 in the modified test paradigm).

In calculating DPOAE signal and noise levels from ${\boldsymbol{z}}$, a weighting strategy was used to reduce or remove the influence of any elements contaminated by intermittent noise. For relatively long signals such as these nine-second sweeps, this strategy allows effective artifact control at the resolution of single analysis frames (500 ms), avoiding the need to discard entire sweeps. An iterative bisquares reweighting algorithm [34] was applied to the magnitudes of the 24 complex coefficients in ${\boldsymbol{z}}$ to obtain a column vector of corresponding weights ${\boldsymbol{w}}$. This method minimizes the weighted sum of squares, with the weight assigned to each data point dependent on the distance of that point from the weighted mean in the previous iteration. Data points near the weighted mean receive full weight, while data points farther from the weighted mean receive reduced weights. The process of reweighting is repeated iteratively until the weighted mean converges (i.e., the fractional change in the current weighted mean from the previous weighted mean reaches some criterion amount). The final weighted mean minimizes the effect of outliers. For ease of subsequent computation, the final weights were normalized by dividing each element of ${\boldsymbol{w}}$ by the sum of ${\boldsymbol{w}}$, so that the weights summed to one. The DPOAE signal pressure was then calculated as the weighted complex mean across sweeps:

4 $$\overline{{z}}={\boldsymbol{z}}{{\boldsymbol{w}}}^{\mathsf{T}},$$

where superscript $\mathsf{T}$ is the transpose operator. For creating GFs, DPOAE (weighted) mean magnitude was calculated as

5 $$\tt{z}=\sqrt{\mathrm{R}\mathrm{e}{\left(\overline{\it{z}}\right)}^{2}+\mathrm{I}\mathrm{m}{\left(\overline{\it{z} }\right)}^{2}},$$

where $\tt{z}$ written in Latin font indicates the variable is strictly real. The DPOAE noise floor was calculated as the weighted estimator of the standard deviation of the mean (the “standard error of the mean”) across sweeps:

6 $${\tt{s}}_{\tt{z}}=\sqrt{\left(\frac{{M}^{-1}}{1-{M}^{-1} }\right)\sum {\boldsymbol{w}}\left(\left({\boldsymbol{z}}-\overline{{z} }\right){\left({\boldsymbol{z}}-\overline{{z} }\right)}^{\mathbf{*}}\right)},$$

where $\overline{{z} }$ is the weighted complex mean of ${\boldsymbol{z}}$, and the asterisk in Eq. 6 indicates complex conjugation. ${\boldsymbol{w}}$ is the normalized vector of weights described above, and M is the number of sweeps.^1^ In Eq. 6, the vectors are multiplied element-wise.

The analysis procedure described above was applied to the 71 analysis frames in the recorded sweep, centered at target values of *L*_2_ ranging from 0 to 70 dB FPL. In Eqs. 5 and 6, the variables $\tt{z}$ and ${\tt{s}}_{\tt{z}}$ refer to values obtained from a single analysis frame. To be clear: ${\boldsymbol{z}}$ is a complex vector across sweeps at a single stimulus level, the scalar $\tt{z}$ is the mean magnitude of ${\boldsymbol{z}}$, and throughout the rest of this paper, ${\bf{z}}$ is a real vector composed of $\tt{z}$ at each analyzed stimulus level. Similarly, the real vector ${\bf{s}}_{\bf{z}}$ is composed of ${\tt{s}}_{\tt{z}}$ across stimulus levels. In other words, ${\bf{z}}$ and ${{\bf{s}}}_{{\bf{z}}}$ are 71 × 1 vectors obtained by analyzing the entire recording. Where needed, subscripts are used to indicate single elements in ${\bf{z}}$ (e.g., ${{\bf{z}}}_{i}$). The best point estimate of the noise floor was taken as the mean across ${{\bf{s}}}_{{\bf{z}}}$, written $\overline{{\tt{s}}_{\tt{z}}}$. To create an GF, ${\bf{z}}$ was plotted as a function of *f*_2_ stimulus magnitude.

**Fig. 1:**
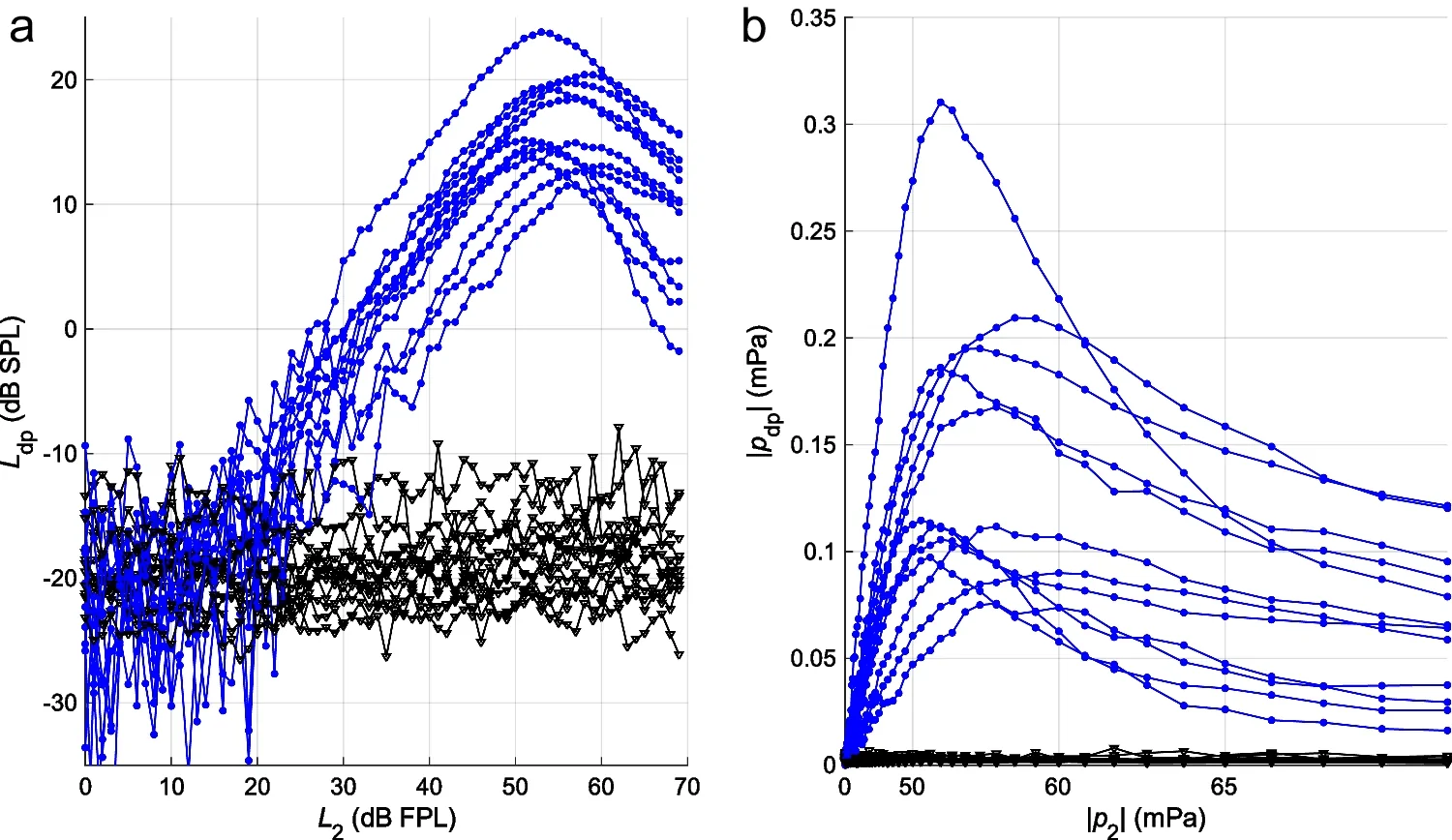
DPOAE GFs for 12 participants. Panel **a** shows data on a log-log scale; Panel **b** shows the same data on a linear-linear scale. Both Panels: Mean DPOAE magnitudes (${\bf{z}}$) are shown by blue lines with dot markers. Noise floors, calculated as the standard error of the mean (${{\bf{s}}}_{{\bf{z}}}$), are shown by black lines with downward pointing triangle markers. Stimulus levels directly measured at the probe tip can be inaccurate due to standing waves in the canal; therefore, in Panel a, measured DPOAE levels (*L*_dp_) are expressed as a function of target *L*_2_ values in dB FPL instead of measured *L*_2_ values in dB SPL. In Panel **b**, the quantities are expressed as linear pressure magnitudes (${|p}_{dp}|$ and ${|p}_{2}|$)

The GFs obtained from 12 participants using the standard swept paradigm are shown in Fig. 1. Figure 1a shows the data on log-log axes, which is helpful for visualizing the lower *L*_2_ levels. Figure 1b shows the same data on linear-linear axes, which is the form in which the data were analyzed and fitted. The linear scale clearly shows the compressive growth of the GF. In the fixed *L*_1_ paradigm, GFs grow to a peak and then decrease. The decrease in the GFs at higher *L*_2_ likely occurs because of two-tone suppression effects and/or changes in the way scattered wavelets combine inside the cochlea as *L*_2_ approaches *L*_1_ [14, 35]. In general, GFs appeared to decrease asymptotically above the peak. For some participants, the GF continued its downward trajectory for *L*_2_ > 65 dB FPL; however, for other participants, the GF became flatter, or in some cases began to increase slightly. The cause of these differences is unclear, and additional experiments with *L*_2_ going to higher levels are needed to understand these observations. For the remainder of this report, GF data are considered for *L*_2_ from 0–65 dB FPL.

### Latent Growth Statistical Model

**Fig. 2:**
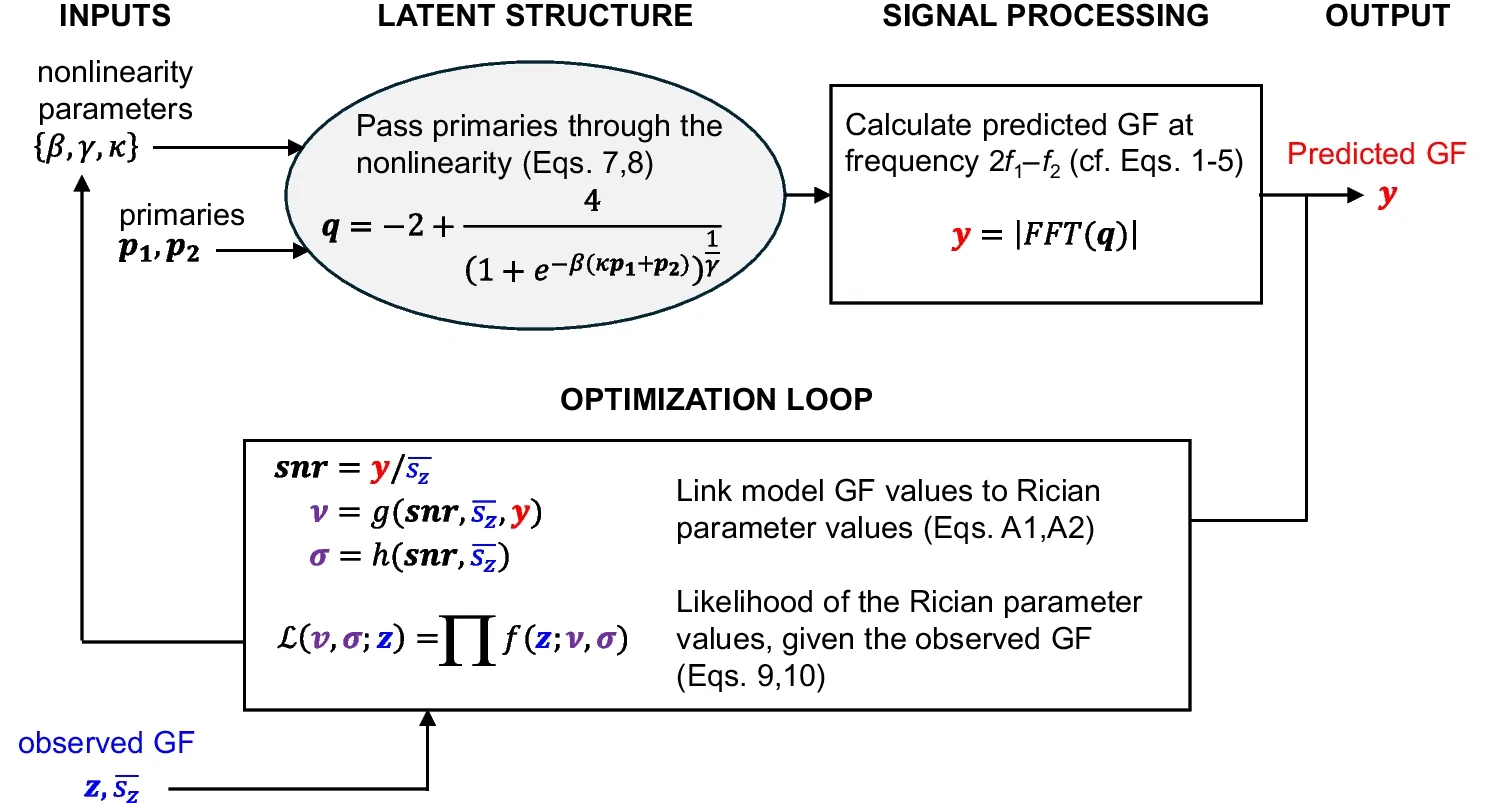
Block diagram of the structural model. The primary input to the model is the variable triple of parameter values for the latent non-linearity ($\{\beta$, $\gamma ,\kappa \}$). A secondary input is the fixed pair of primary (stimulus) waveforms used to evoke DPOAEs (${{\boldsymbol{p}}}_{1},{{\boldsymbol{p}}}_{2}$). The primary waveforms are passed through the nonlinearity, resulting in a distorted waveform (${\boldsymbol{q}}$), which is analyzed to extract magnitude at the 2*f*_1_-*f*_2_ frequency (${\boldsymbol{y}}$, shown in red font). The vector ${\boldsymbol{y}}$ is the output of the model and is referred to as the “predicted GF”. An iterative loop is used to adjust the nonlinearity parameter values to produce the optimal match between the predicted GF and the observed GF data measured from an individual ear (${\bf{z}},\overline{\bf{s}_{\bf{z}}}$, shown in blue font). Optimization is based on maximum likelihood. A generalized linear regression model is used, wherein each GF value is conceptualized as a random draw from the Rician distribution. Rician parameter values are dependent on SNR, which varies across the GF. The Rician parameter values ($\{{\boldsymbol{\nu}},{\boldsymbol{\sigma}}\}$, shown in purple font) associated with each element in the predicted GF (i.e., each element in vector ${\boldsymbol{y}}$) are determined using link functions. The likelihood of $\{{\boldsymbol{\nu}},{\boldsymbol{\sigma}}\}$, given the observed GF (${\bf{z}}$) is calculated. For an observed GF measured from an individual ear, the optimal values for the latent nonlinearity triple are those producing the predicted GF with corresponding Rician parameters having the maximum joint probability, given the observed GF

It is of interest to characterize observed GFs with a statistical model. Models reduce the dimensionality of the data, allowing a succinct description using a relatively small number of parameters. They also facilitate quantifying GF characteristics, such as slopes and thresholds, which have been used to assess cochlear function. Under the assumption that DPOAEs are causally related to an underlying nonlinear cochlear vibration pattern, we hypothesized that the observed GFs could be characterized by a small number of parameters belonging to a compressive nonlinear growth function representing this vibration pattern. This nonlinear growth function is assumed to correspond to a physical reality (vibration of cochlear structures) that could, in principle, be measured but currently cannot be noninvasively observed in humans. We constructed a latent growth statistical model which related the set of unobservable latent variables (the non-linearity parameters) to observable variables (the GF and noise floor estimate) via a set of structural equations and a generalized linear regression. A block diagram of the model is shown in Fig. 2. The latent portion of the model is shown inside the ellipse. The stimuli (DPOAE primaries) were passed through the non-linearity, and the output of the non-linearity was analyzed in the frequency domain to obtain a resulting predicted GF. The optimization loop shows the process by which the values of the coefficients for the non-linearity were adjusted to produce the predicted GF that best fitted the observed GF data.

There are multiple sources of cochlear non-linearity that may contribute to DPOAE generation, including processes associated with mechanoelectrical transduction (e.g., [36]) and electrical-to-mechanical transduction (e.g., [37]). The non-linearity used in the structural model does not necessarily represent any of the specific non-linearities associated with DPOAE generation, but it may approximate a composite non-linearity that captures the effective contribution of individual mechanistic non-linearities. Previous work has described the conversion of sound stimuli into cochlear responses using a variety of saturating non-linear functions with sigmoidal shapes, such as arctangent, hyperbolic arctangent, logistic, first- or second-order Boltzmann, and third-order polynomials [21–27]. We used a simplified form of the generalized logistic function [38] as the non-linearity for the latent portion of the statistical model.

The generalized logistic function has six free parameters, providing great flexibility. In preliminary analyses, we found that we could accurately fit our observed GF data using a modified version of the generalized logistic function, wherein the number of free parameters was reduced to two (β and γ), plus one additional stimulus scaling parameter κ. The simplified generalized logistic function had the form

7 $${\boldsymbol{q}}=-2+\frac{4}{{\left(1+{e}^{-\beta {\boldsymbol{p}}}\right)}^{\frac{1}{\gamma }}} ,$$

8 $${\boldsymbol{p}}=\kappa {{\boldsymbol{p}}}_{1}+{{\boldsymbol{p}}}_{2} .$$

**Fig. 3:**
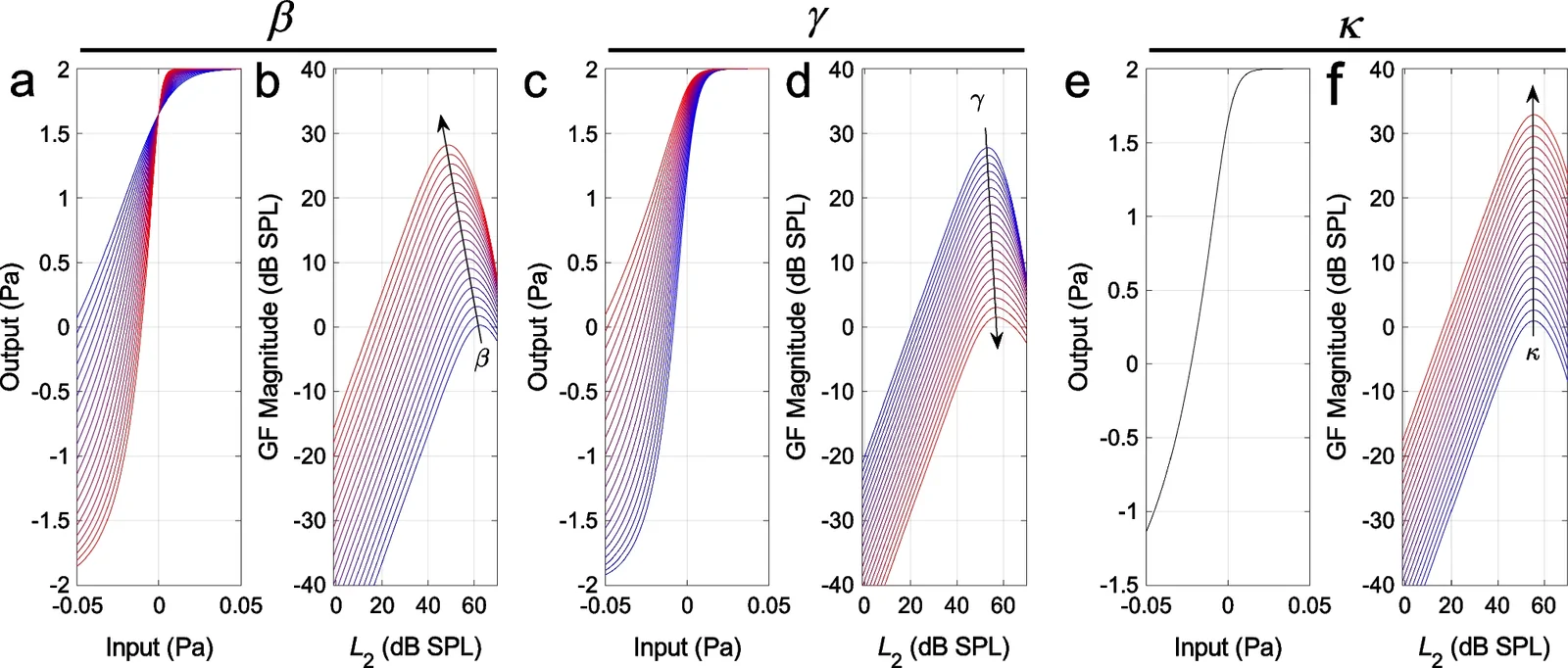
Effects of the three parameters on the shape of the non-linearity (Panels **a**, **c**, and **e**) and the shapes of the resulting predicted GFs (Panels **b**, **d**, and **f**). The effects of each parameter are shown in pairs of panels. Panels **a**, **b** show the effects of changing β with the other parameters held constant. Likewise, Panels **c**, **d** and Panels **e**, **f** show the effects of changing γ and κ, respectively, with the other parameters held constant. When held constant, the values used were the mean of the fitted values from the 12 participants in this study (Table 1). In all panels (except **e**), curves are shown for 20 selected values of the parameter of interest. Increasing values of the parameters are indicated by colors changing from blue to red, and the main directions of GF change are indicated by arrows. Panels** a**, **b** show the effect of increasing the value of β is to steepen the slope of the nonlinearity (**a**) and move the peak of the GF up and to the left (**b**). Panels **c**, **d** show the effect of increasing the value of γ is to move the location of maximum growth towards the upper asymptote of the nonlinearity (**c**) and move the peak of the GF down and to the right (**d**). Panels **e**, **f** show that κ has no effect on the nonlinearity since it does not appear in Eq. 7 (**e**); however, the effect of increasing the value of κ is to move the entire GF up (**f**), i.e., expressed on a log–log scale, the GF simply starts and ends at a higher y-axis value with increasing κ, while the shape remains constant

In Eq. 7, the scalar values −2 and 4 set the minimum and maximum outputs of the function at ± 2. These values were chosen for convenience in working with acoustic data (representing 2 Pa, or 100 dB SPL, a realistic range of sound pressures), but the goodness of the obtained fits is not dependent on these specific values. Output of the non-linearity was a function of instantaneous stimulus sound pressure (${\boldsymbol{p}}$). The vector ${\boldsymbol{p}}$ was the summed pressures of the two primaries, ${{\boldsymbol{p}}}_{1}$ and ${{\boldsymbol{p}}}_{2}$ (having frequencies ${f}_{1}$, ${f}_{2}$ and levels ${L}_{1}$, ${L}_{2}$, respectively). The third free parameter, κ, scaled the lower frequency primary prior to summing with the higher frequency primary, as shown in Eq. 8. The waveform outputs of the instantaneous nonlinearity (${\boldsymbol{q}}$; Eq. 7) were converted to the frequency domain, and the magnitudes at frequency 2*f*_1_–* f*_2_ were used to create a predicted GF (${\boldsymbol{y}}$). Figure 3 shows the effects of the three parameters on the nonlinearity (Panels a, c, and e) and the 2*f*_1_–* f*_2_ GF (Panels b, d, and f). With regard to the nonlinearity, the parameter β controls the steepness of growth, and the parameter γ controls where maximum growth occurs (γ >1 moves the maximum growth towards the upper asymptote).The parameter κ has no effect on the nonlinearity itself, and its main effect is to linearly increase or decrease the overall GF magnitude.

Producing the predicted GF (${\boldsymbol{y}}$) that best matched an observed GF (${\bf{z}}$) required optimizing the parameter triplet $\{\beta ,\gamma ,\kappa \}$. The parameter values were estimated using likelihood and the probability density function (PDF) of the Rician distribution. GFs are generally obtained in such a way (i.e., the averaging time is held constant for each stimulus level) that the DPOAE magnitude varies while the underlying noise floor remains stationary. As a result, SNR varies along the x-axis. If all data points in the GF have an SNR greater than approximately 10 dB, the signal + noise magnitude can be modeled statistically using the Normal distribution. However, when DPOAEs have less than approximately 10 dB SNR, the associated signal + noise magnitude is no longer normally distributed. In the limit of no DPOAE present, the signal + noise magnitude is Rayleigh distributed. At low SNRs (i.e., $\mathrm{S}\mathrm{N}\mathrm{R}\lesssim 10 \mathrm{d}\mathrm{B})$, the signal + noise magnitude varies systematically between the Rayleigh and Normal distributions, and the Rician distribution quantifies this changing distribution. The Rician distribution is well known in various fields, including the signal processing of noisy images, such as those obtained by MRI. It has been occasionally used to study otoacoustic emissions (e.g., [39]).

Formally, the Rician distribution describes the magnitudes of circular-symmetric bivariate normal random variables, including those with non-zero means. Expressed in terms of the variables used in this paper, the PDF of the Rician distribution is

9 $$f\left(\tt{z};\nu,\sigma \right)=\frac{\tt{z}}{{\sigma }^{2}}{e}^{\left(-\left({\tt{z}}^{2}+{\nu}^{2}\right)/2{\sigma }^{2}\right)}{I}_{0}\left(\frac{\tt{z}\nu}{{\sigma}^{2}}\right),$$

where $f\left(\tt{z};\nu,\sigma \right)$ is the probability density of the DPOAE magnitude ($\tt{z}$), given the Rician parameters ν and σ. ${I}_{0}$ is the modified Bessel function of the first kind with order zero. The distribution has two parameters: ν, which controls the location, and σ, which controls the scale (spread). To use the Rician distribution in the context of fitting predicted GFs to observed data, additional functions are needed to link each predicted GF value (each element of ${\boldsymbol{y}}$) to its Rician parameter values, given the expected SNR. In other words, the linking functions were needed because the parameter values of the Rician distribution depend on SNR, which in this study varied as a function of *L*_2_. Given the predicted GF value (“signal”) and the measured noise floor (“noise”) at a given *L*_2_, the linking functions returned the appropriate SNR-based Rician parameter values. We defined two functions mapping SNR to the parameter values of the Rician distribution. Details are given in Appendix A. Applying these functions to ${\boldsymbol{y}}$ yielded two vectors ${\boldsymbol{\nu}}$ and ${\boldsymbol{\sigma}}$, with elements which correspond both to ${\boldsymbol{y}}$ and the associated vector of primary levels. Maximum likelihood estimation was used to determine the most likely values of vectors ${\boldsymbol{\nu}}$ and ${\boldsymbol{\sigma}}$, given an observed GF. The likelihood function was

10 $$\mathcal{L}\left({\boldsymbol{\nu}},{\boldsymbol{\sigma}};{\bf{z}}\right)=\prod\limits_{i=1}^{N}f({\tt{z}}_{i};{\nu}_{i},{\sigma}_{i}),$$

where $\mathcal{L}\left({\boldsymbol{\nu}},{\boldsymbol{\sigma}};{\bf{z}}\right)$ is the likelihood of the parameter vectors ${\boldsymbol{\nu}}$ and ${\boldsymbol{\sigma}}$, given the observed GF data in the magnitude vector ${\bf{z}}$, and N is the number of analysis frames. Note that although the likelihood is computed in terms of ${\boldsymbol{\nu}}$ and ${\boldsymbol{\sigma}}$, these values were linked deterministically to the predicted GF via SNR, so that the likelihood of the set of coefficients $\{\beta ,\gamma ,\kappa \}$ was indirectly determined.

Maximum likelihood estimation is an optimization problem, wherein the values of the parameters that maximize the joint probability of the data are sought. Numerical solutions can be found using a variety of optimization procedures. In this study, we used MATLAB’s constrained nonlinear optimization function *fmincon* to minimize the negative log likelihood, which is equivalent to finding the maximum likelihood estimate (MLE). The MLE for the parameter values producing the predicted GF was the set of coefficients mapped to the pair of vectors ${\boldsymbol{\nu}}$ and ${\boldsymbol{\sigma}}$ that produced the maximum likelihood value, i.e.,

11 $$\begin{array}{c}\begin{array}{cc}\mathrm{M}\mathrm{L}\mathrm{E}=\underset{{\boldsymbol{\nu,\sigma}}}{\text{arg max}}& \mathcal{L}\left({\boldsymbol{v}},{\boldsymbol{\sigma}};{\bf{z}}\right)\mapsto \end{array} \left\{\widehat{\beta },\widehat{\gamma },\widehat{\kappa }\right\}\\ \end{array}.$$

In summary, when magnitude GFs are the measurement of interest, the signal + noise distribution varies at SNRs below approximately 10 dB, violating the homoskedasticity assumption made by standard regression models. Inclusion of low-SNR data in the GF therefore necessitates using the Rician distribution to model the noise. Each element of the predicted GF was linked to its set of SNR-dependent Rician parameter values, and maximum likelihood estimation was used to find the predicted GF that best fit the observed GF data.

### GF Peak, Inflection Points, and Maximum Slope

The structural equation approach described above yielded estimates for the latent variables associated with the non-linearity. Ideally, the values of these latent parameters ($\widehat{\beta },\widehat{\gamma },\widehat{\kappa }$) are key points of interest for inferences and predictions about measures of clinical interest, such as cochlear nonlinearity, behavioral audiometric thresholds, loudness perception, and detection of subclinical damage. The strength of these relationships remains to be quantified using suitably large data sets, as well as compared with the performance of traditional descriptions of GFs, such as peak levels and slopes. To facilitate comparisons with traditional measures, each predicted GF was quantified at four points: 1) peak, 2) inflection point below the peak, 3) inflection point above the peak, and 4) maximum slope. We found these values by first fitting the predicted GF with a cubic spline (on a linear–linear scale), from which derivatives and their zero crossings could easily be computed.

Some previous work with GFs obtained from other paradigms has estimated a “knee point” or inflection point associated with the onset of cochlear compression (e.g., [40, 41]), defined as the point where the GF departs from approximate linear growth. (Note that some reports focus on GF compression in log–log space, as opposed to linear–linear space.) In the fixed *L*_1_ paradigm, the slope changes continuously throughout the GF (Fig. 1b); however, it does not change at a constant rate. We defined inflection points where the rate of change in slope was maximal (i.e., the location of the minimum and maximum of the second derivative of the spline fit in linear space). The inflection point below the peak was the *L*_2_ value at which the slope was most rapidly changing towards maximum compression. The inflection point above the peak was the *L*_2_ value at which the slope was most rapidly changing away from maximum compression.

In previous work, GFs have sometimes been used to estimate “thresholds”. Estimates have been made in log-log, semi-log, and linear-linear space, with thresholds defined as the lowest stimulus level at which DPOAEs are measurable or the level at which the DPOAEs theoretically become zero based on a mathematical function fit to the data (e.g., [42, 43]). For the fixed *L*_1_ paradigm, this latter concept of threshold does not appear to make sense, since a non-zero GF magnitude is expected for any* f*_2_ stimulus magnitude > 0 Pa, so long as there is some amount of non-linearity associated with cochlear vibration near the *f*_2_ place. Defining “threshold” as the *L*_2_ value where the GF reaches the noise floor is also undesirable, because such a measure conflates the signal and the noise levels. Noise floor varies as a function of frequency, hardware used, and averaging time. Noise floor also varies across individual subjects even when accounting for these other variables. In our sample of 12 participants, for example, noise floors showed a 10.7 dB range (Fig. 1a). Previous studies have rightly considered SNR when fitting GF magnitudes, for example including only data points with SNR > 6 dB ([e.g., 42, 43]). Such SNR-based criteria, like the Rician-based method described in this paper, improve the robustness of estimation procedures.

Based on theoretical expectations as well as human and animal measurements made in our labs, subjects with pristine hearing have steeper maximum GF slopes (on a linear-linear plot), while those with hearing loss have shallower slopes. In the fixed *L*_1_ paradigm, the steepest slope appears to be found in the limit as *f*_2_ stimulus magnitude (${|p}_{2}|$) approaches 0 Pa. Support for this assertion will be presented in a later section. We therefore defined GF maximum slope as the first derivative of the spline fit evaluated at ${|p}_{2}|$ = 0 Pa. Although this GF maximum slope is not a “threshold” in the traditional sense, it may correlate with behavioral audiometric thresholds in a manner similar to GF “thresholds” reported by previous work. Though it is not possible for a threshold measure to be truly independent of noise floor (since noise will always affect the precision of the DPOAE magnitude estimate), the slope of the predicted GF in the limit as ${|p}_{2}|$ approaches 0 Pa is otherwise independent of noise floor.

### Interval Estimates

Confidence intervals (CIs) may be obtained in several ways. When evaluating the present GF data, we found that the commonly-used profile likelihood approach underestimated the actual variability, likely due to heteroskedastic residuals and relatively modest sample sizes. To obtain accurate interval estimates, we computed bootstrap confidence intervals. The paired bootstrap and residual bootstrap [44] are techniques commonly applied to regression models, but these are inappropriate in the present case due to nonconstant spacing on the x-axis and the presence of heteroskedasticity. In such cases, the wild bootstrap is an option [45, 46]. We implemented a modified version of the wild bootstrap which involved adding random samples of noise to the model fit, rather than adding reweighted versions of the residuals themselves. For each resample, ${\widetilde{\tt{z}}}_{i}={\widetilde{y}}_{i}+{\widetilde{\zeta }}_{i}$, where the tilde accents indicate resampled values. ${\widetilde{\zeta }}_{i}$ was a complex number generated by the sum of two random draws from a normal distribution centered at zero with a standard deviation of $\overline{{\tt{s}}_{\tt{z}}}\sqrt{M/2}$, with the second draw multiplied by $\sqrt{-1}$ to make ${\widetilde{\zeta }}_{i}$ complex. This resampling was done for M sweeps, so the resulting matrix of resampled values could be processed as described by Eqs. 4–6. The resampled vectors $\widetilde{{\bf{z}}}$ and $\widetilde{{{\bf{s}}}_{{\bf{z}}}}$ were fit as described above to obtain resampled MLE estimates $\{\widetilde{\beta },\widetilde{\gamma },\widetilde{\kappa }\}$. Using these resampled coefficients, predicted GF curves were generated, and their associated slopes, peaks, and inflection points were found. One hundred bootstrap resamples were obtained, and 95% confidence intervals (CIs) were computed from the sorted resamples for each measurement of interest. One hundred resamples is generally too small for determining statistical significance; however, in the present case the purpose of the resamples was to give a sense of the precision associated with the fits and measurements for each participant, for which 100 resamples was sufficient.

## Results

### Obtained Fits

**Fig. 4:**
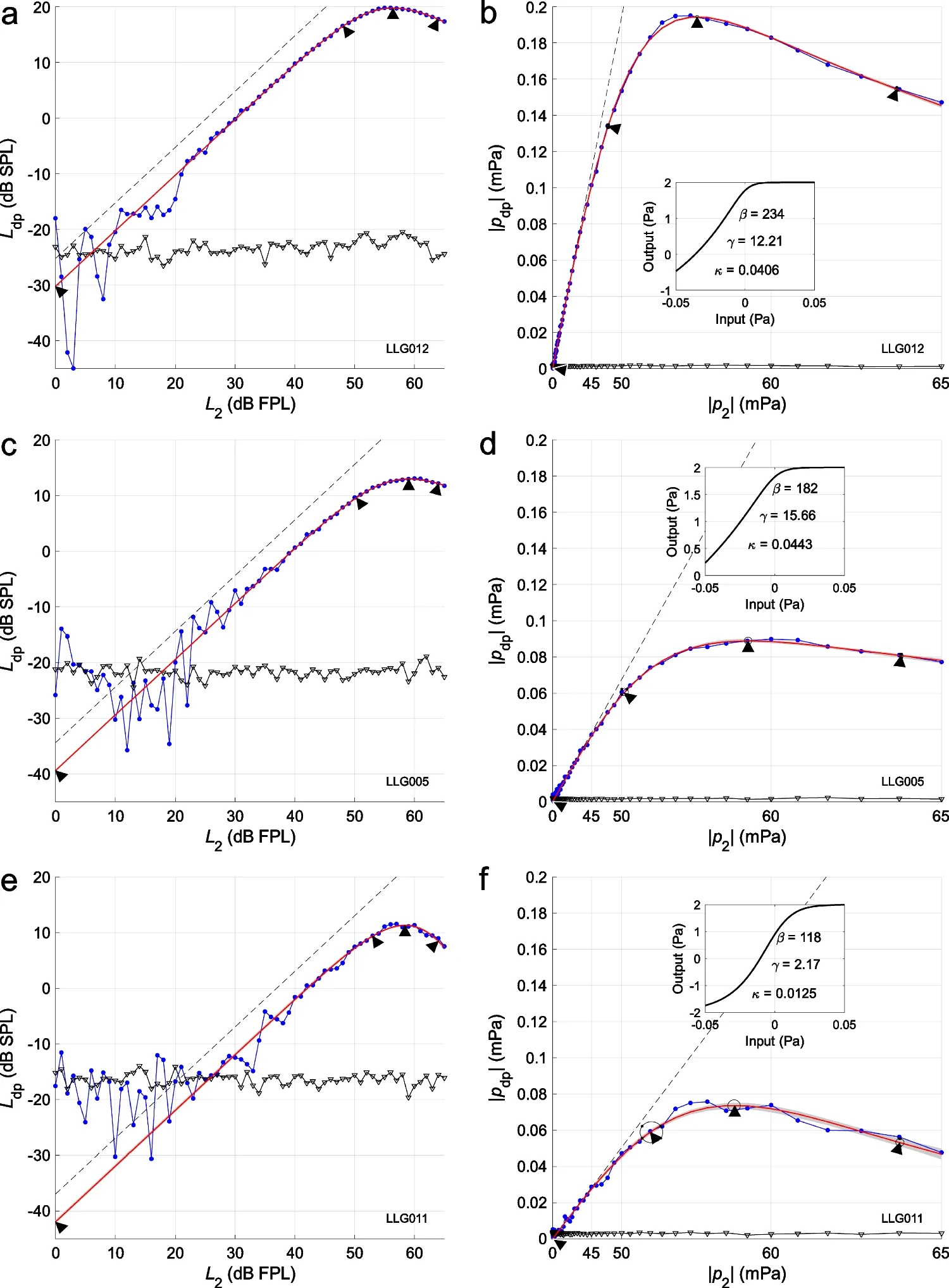
Example GFs and fits. Each row shows one participant. Left column shows data plotted on log-log axes. The dashed diagonal line in each panel shows the slope of linear growth. Right column shows the same data plotted on linear-linear axes. The dashed line shows linear growth. In each panel, an inset plot shows the shape of the non-linearity that produced the predicted GF, along with the parameter values. The x-axis (input) range of the non-linearity is constrained to ± 0.05, corresponding to ~ 67 dB SPL in the model. In all panels, DPOAE magnitudes ($\bf{z}$) are shown by blue lines with dot markers. Noise floors (${\bf{s}}_{\bf{z}}$) are shown by black lines with downward pointing open triangle markers. Red lines show the predicted GF. The bootstrapped 95% CI of each predicted GF is shown by surrounding gray shading; however, on the large axes ranges of these plots, the CI shading is only readily apparent in Panel** f**. Filled black triangles point to the locations of the peak, inflection points, and maximum slope of the predicted GF. Bootstrapped 95% CIs for each of these points are shown by an open black circle around the vertex of the triangle. On the large axes ranges of these plots, the CIs are only readily visible in panels **d** and **f**

All 12 participants contributed usable data, and no recordings were discarded. GF peak amplitudes (i.e., the maximum amplitude in each GF) ranged from 11.3 to 23.6 dB SPL (mean = 16.5 dB SPL, SD = 3.67). GF noise floors (averaged across *L*_2_) ranged from −23.5 to −12.8 dB SPL (mean = −18.9 dB SPL, SD = 3.1), and peak SNRs ranged from 27.8 to 43.2 dB (mean = 35.4 dB, SD = 3.8). All GFs were well fit using the latent growth model. Representative fits to GFs obtained from three participants are shown in Fig. 4. In each row, data are shown for a single participant in log space (left panel) and in linear space (right panel). The observed GFs are shown by connected filled blue circles, and the predicted GFs are shown with overlaid smooth red curves. The locations of peaks, inflection points, and maximum slopes are pointed to by filled black triangles. The noise floors are shown by connected down-pointing unfilled triangles. 95% CIs are shown by gray shading surrounding the predicted GFs and by open black circles surrounding the peaks, inflection points, and maximum slopes. Because overall SNRs were high, the CIs were small and are difficult to see on the large axis ranges of these plots. The participant with the poorest SNR and largest CIs is shown in Fig. 3e, f. The CIs for this participant are clearly visible in the linear plot (Panel F). Table 1 summarizes the group results for the various measures that were obtained.

**Table 1:** Group-level summary of fitting results. See section entitled “GF Peak, Inflection Points, and Maximum Slope” for details. “Inflection 1” refers to the inflection point below the peak of the GF, and “Inflection 2” refers to the inflection point above the peak. “Peak Width” was computed as the distance between the location of the inflection points. “Peak Height” was computed as the distance between peak amplitude and the average of the two inflection point amplitudes

	Mean	SD	Min	Max
$\widehat{\beta }$	234.20	88.93	117.89	439.97
$\widehat{\gamma }$	7.62	4.87	1.22	16.27
$\widehat{\kappa }$	0.0233	0.0120	0.0075	0.0443
Max slope	0.0253	0.0132	0.0080	0.0579
Max slope (dB)	−33.03	4.71	−41.95	−24.75
Peak location (*L*_2_)	55.39	2.74	51.47	59.03
Peak amplitude (dB SPL)	16.47	3.67	11.32	23.58
Peak width (dB)	13.90	1.45	11.02	15.84
Peak height (dB)	2.70	0.47	2.11	3.87
Inflection 1 location (*L*_2_)	48.23	2.94	42.68	52.99
Inflection 1 amplitude (dB SPL)	13.75	3.63	9.38	21.21
Inflection 1 slope	0.0148	0.0077	0.0042	0.0325
Inflection 2 location (*L*_2_)	62.13	2.16	58.17	64.04
Inflection 2 amplitude (dB SPL)	13.80	3.85	8.48	19.72
Inflection 2 slope	−0.0030	0.0020	−0.0085	−0.0007

It is helpful to keep in mind that on linear-linear plots, the growth near ${|p}_{2}|$= 0 Pa manifests as different slopes across participants, whereas this same growth on log–log plots manifests primarily as different y-axis offsets. Therefore, it is expected that participants with hearing loss will exhibit shallower GF slopes on linear–linear plots, while on log–log plots they will show very similar slopes but have different y-axis offsets. This distinction is important when considering whether different types of hearing loss alter GF slope—the answer may depend on which space (i.e., linear versus log) an observer is referring to.

Table 2 shows the sample Pearson correlation coefficients associated with the non-linearity parameters and the traditional GF measures. Due to the small sample size (12 participants), statistical significance is not reported. Rather, to highlight potentially important relationships, coefficients with values larger than 0.5 are shown in bold font. The choice of 0.5 as a cutoff is somewhat arbitrary but was chosen to highlight relationships that are likely to be related. Future analyses with larger sample sizes are needed to accurately quantify these relationships. Based on this sample, the parameter estimates $\widehat{\gamma }$ and $\widehat{\kappa }$ were strongly related to each other (*r* = 0.70), but less strongly related to the parameter estimate $\widehat{\beta }$ (*r* = 0.48 and −0.18, respectively). The parameter estimate $\widehat{\beta }$, which controls the steepness of nonlinearity growth, was strongly associated with the *L*_2_ locations of the peak and inflection points (*r* = −0.82, −0.97, −0.84), but not related to the GF amplitudes themselves. Of the three parameters, only β was related to the maximum GF slope. The parameter estimate $\widehat{\gamma }$, which controls the stimulus level at which maximum growth occurs in the nonlinearity, was most strongly related to GF peak height (distance between peak amplitude and the average of the two inflection point amplitudes; *r* = 0.68) and GF peak width (distance between the location of the inflection points; *r* = −0.59), but not related to the peak and inflection amplitudes themselves. Finally, the parameter estimate $\widehat{\kappa }$, which increases or decreases the overall GF magnitude, was related to peak *L*_2_ location and overall GF peak height (*r* = 0.61, −0.63), as well as the location and amplitude of the second inflection point (*r* = 0.61, 0.57).

**Table 2:** Pearson correlation coefficients for the nonlinearity parameter estimates and GF measures. “Inflection 1” refers to the inflection point below the peak, and “Inflection 2” refers to the inflection point above the peak. “Peak Width” was computed as the distance between the location of the inflection points. “Peak Height” was computed as the distance between peak amplitude and the average of the two inflection point amplitudes. Coefficients > 0.5 are shown in bold font (see text for details)

	$\widehat{\beta }$	$\widehat{\gamma }$	$\widehat{\kappa }$
$\widehat{\beta }$	1	0.48	−0.18
$\widehat{\gamma }$	0.48	1	**0.70**
$\widehat{\kappa }$	−0.18	**0.70**	1
Max slope	0.42	0.01	−0.03
Max slope (dB)	**0.55**	0.08	−0.01
Peak location (*L*_2_)	**−0.82**	0.04	**0.61**
Peak amplitude (dB SPL)	0.01	−0.02	0.33
Peak width (dB)	**0.71**	**0.68**	0.39
Peak height (dB)	0.04	**−0.59**	**−0.63**
Inflection 1 location (*L*_2_)	**−0.97**	−0.36	0.26
Inflection 1 amplitude (dB SPL)	−0.06	−0.16	0.22
Inflection 1 slope	0.48	0.10	0.04
Inflection 2 location (*L*_2_)	**−0.84**	−0.04	**0.61**
Inflection 2 amplitude (dB SPL)	0.07	0.26	**0.57**
Inflection 2 slope	−0.05	0.46	0.32

### Verification of Predictions for Low Stimulus Levels

**Fig. 5:**
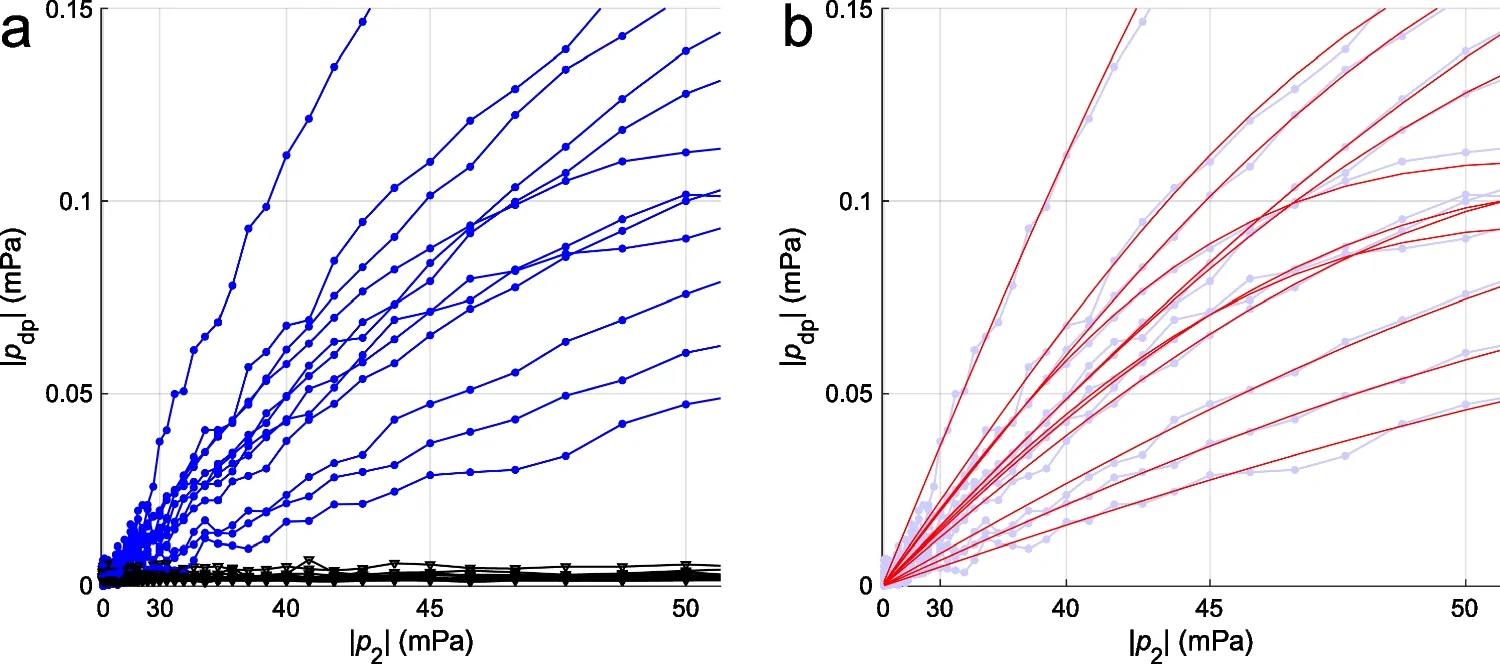
Growth at lower *f*_2_ stimulus magnitudes. DPOAE GFs for 12 participants are shown on linear-linear axes. Plots are shown on truncated axes to focus on lower portions of the GFs (*L*_2_ < 50 dB FPL). Panel **a** shows GF data. Mean DPOAE magnitudes ($\bf{z}$) are shown by blue lines with dot markers. Noise floors (${\bf{s}}_{\bf{z}}$), are shown by black lines with triangle markers. Panel **b** shows the same DPOAE magnitude data in light gray, overlayed by predicted GFs (red curves)

The previous section showed results obtained using a latent growth model utilizing a compressive nonlinearity coupled with a Rician model of the noise and maximum likelihood estimation. This method produced predicted GFs for *f*_2_ stimulus magnitudes from ~ 0.0356 Pa (*L*_2_ = 65 dB FPL) down to 0 Pa. Portions of the predicted GFs that fall below the noise floor can be considered predictions of the magnitudes and growth of DPOAEs that are hidden by the noise. The accuracy of these predictions relies on the following assumptions about how GFs behave in the fixed *L*_1_ paradigm: 1) When* f*_2_ is absent (i.e., *f*_2_ stimulus magnitudes = 0 Pa), the DPOAE magnitudes associated with all *L*_1_ are negligibly small, approaching zero. From a theoretical perspective, the intermodulation distortion that creates the DPOAEs arises from the presence of two or more stimulus tones interacting in a nonlinear cochlea. When only one tone is present, no intermodulation distortion is produced, (except for negligible amounts of background distortion due to Brownian motion and other intracochlear “noise”). Conversely, whenever the cochlea is vibrating in a nonlinear manner (as would be the case when *L*_1_ is fixed at a moderate level, such as 65 dB FPL in the current study), the GF will have some non-zero value for all *f*_2_ stimulus magnitudes > 0 Pa. 2) The underlying GF (i.e., the GF in the absence of variability from random noise) plotted on a linear scale varies smoothly and monotonically between zero and its peak value. This assumes the absence of notches, such as may occur due to two-source (distortion versus reflection) interference. Based on measurements in our labs, in the fixed *L*_1_ paradigm, the occurrences of such notches are rare so long as test frequencies are not chosen at dips in the DPOAE fine structure. Figure 5 provides empirical support for these assumptions. GFs for each participant are plotted from 0 Pa to just below the peak (0.0063 Pa; 50 dB FPL). Figure 5a shows the data only, and Fig. 5b shows the predicted GFs overlayed on the data.

We hypothesized that the predicted GFs accurately predict DPOAE levels below the swept paradigm noise floor. We tested this hypothesis using a modified paradigm in which the standard swept measurements were interleaved with two minutes of averaging at a single low *L*_2_ level of 5 dB FPL (details given in the Test Paradigms section). The long averaging time at a single level reduced the noise floor down to approximately −40 dB SPL, revealing the DPOAE magnitude at this level and allowing comparison with the level expected based on the predicted GF. For this portion of the study, a DPOAE SNR > 10 dB at *L*_2_ = 5 dB FPL was chosen as the criterion for inclusion in the analysis. While 3 or 6 dB are commonly used cutoff values, these are most appropriate for determining the presence or absence of a signal. In the present case, the focus was on accuracy of prediction, making 10 dB a more appropriate criterion. The data for 9 of the original 12 participants met this criterion. For these nine participants, the mean SNR at *L*_2_ = 5 dB FPL was 15.16 dB (SD = 3.09, min = 12.28, max = 20.67).

**Fig. 6:**
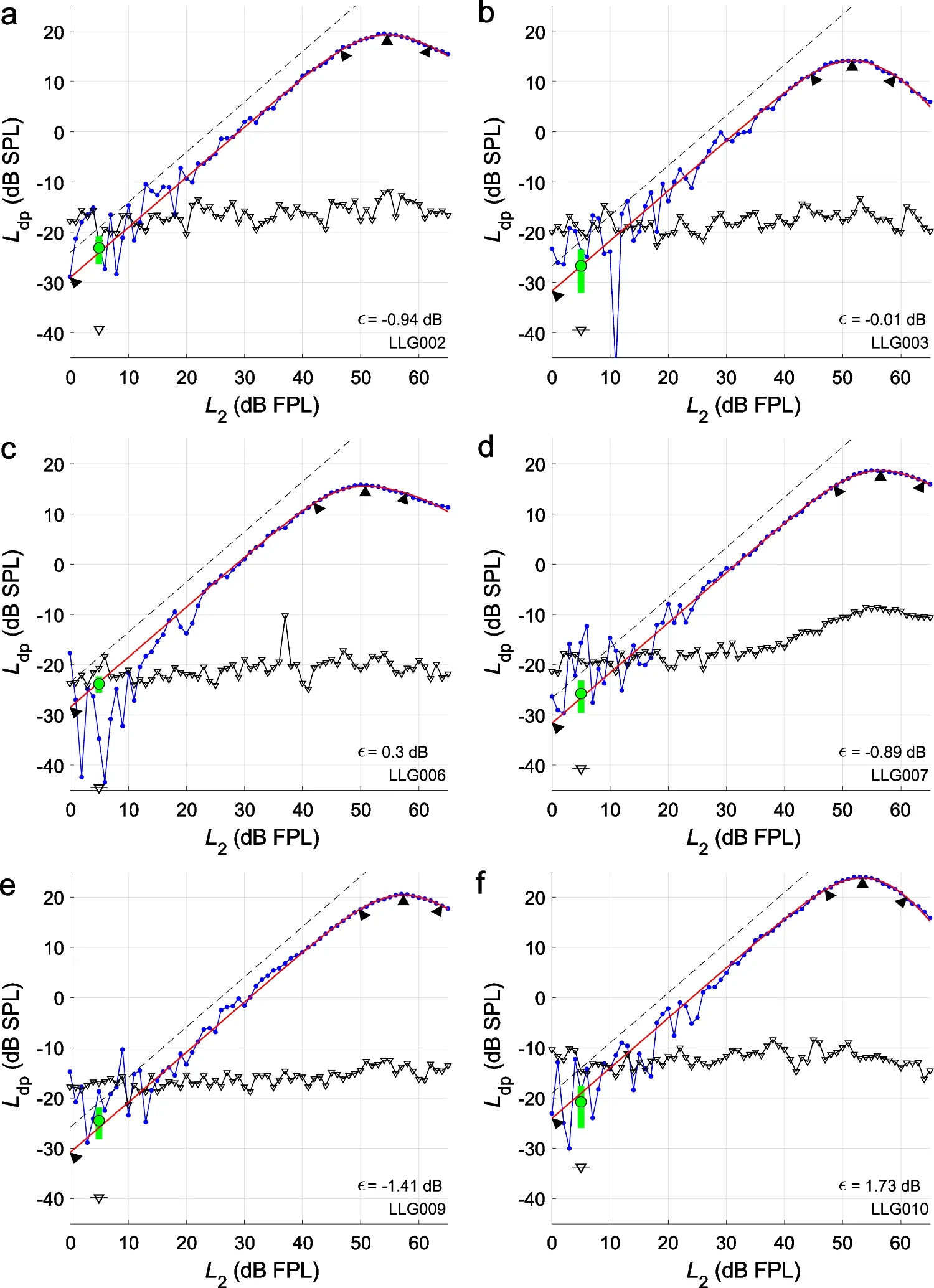
Examples of predicted versus measured DPOAE magnitude at a low stimulus level (*L*_2_ = 5 dB FPL). Each panel shows one participant. Participant numbers are shown in the lower right of each panel, along with the prediction error (ϵ). DPOAE magnitudes ($\bf{z}$) are shown by blue lines with dot markers. Noise floors (${\bf{s}}_{\bf{z}}$) are shown by black lines with downward pointing open triangle markers. Red lines show the predicted GF. Measured DPOAE magnitudes at *L*_2_ = 5 dB FPL (after 26 min of averaging) are shown by green circles with black outlines. Green vertical bars show the 95% confidence interval around each point estimate. The open black triangle with a horizontal line through it (located directly beneath each green circle) shows the associated noise floor after 26 min of averaging

Six representative examples are shown in Fig. 6, panels a, b, c, d, e, and f. Visually, the data showed a close match between predicted and measured levels at *L*_2_ = 5 dB FPL, as seen by the green circle in each panel falling close to the predicted GF (red line). In all cases, the predicted GF passed through the 95% confidence interval (vertical green bar) around the low-level measurement. The accuracy of the predicted GF at low levels was quantified by subtracting the measured DPOAE magnitude at *L*_2_ = 5 dB FPL from the predicted GF magnitude at the same *L*_2_ stimulus level. The dB difference was taken as the prediction error. The prediction errors appeared to be normally distributed around zero with a mean value of −0.05 dB (SD = 1.88, min = −3.43, max = 2.89). The mean prediction error was not statistically different from zero (*t*(8) = −0.0912, *p* = 0.9296). Taken together, the evidence suggests that in the fixed *L*_1_ paradigm, the predicted GFs are an accurate indicator of DPOAE magnitude at low *L*_2_ stimulus levels, even when those predicted levels are below the standard noise floor.

### Fitting at Poor SNRs

**Fig. 7:**
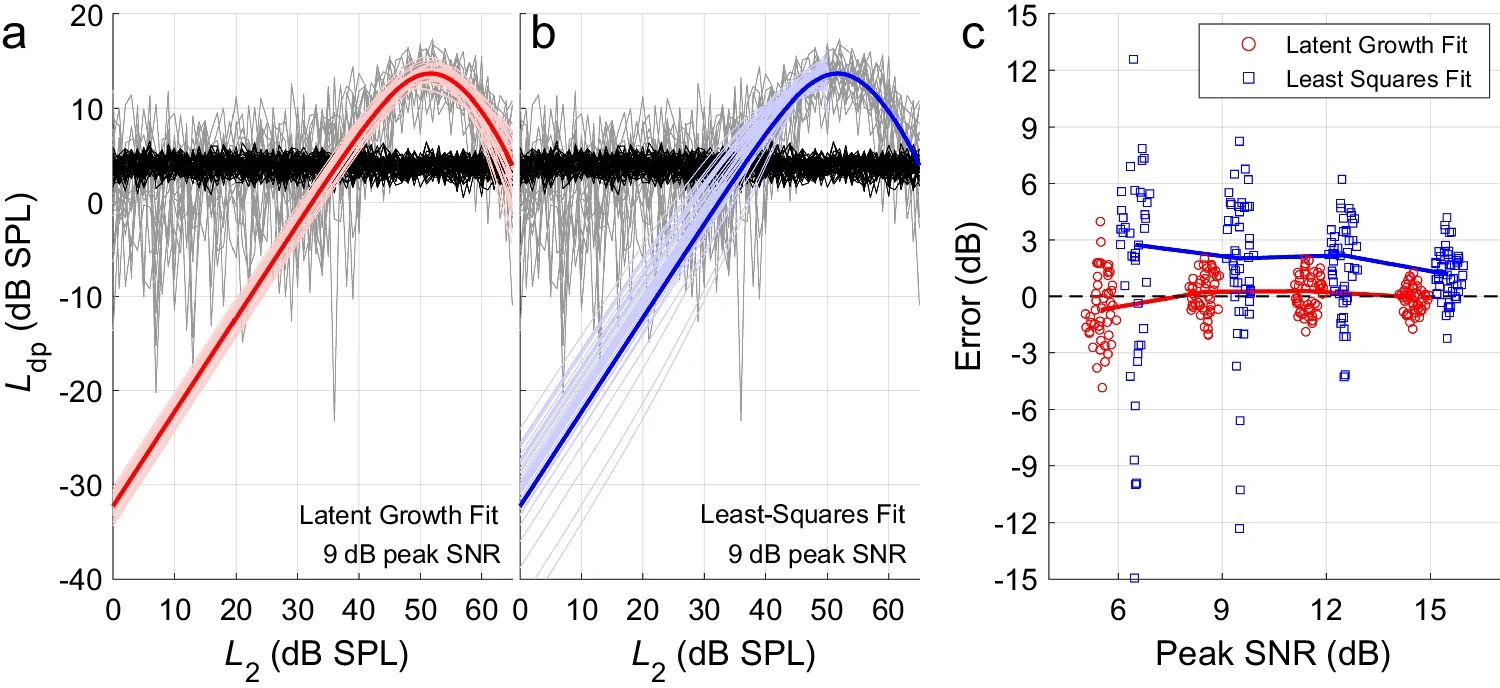
Expected fitting accuracy at poor SNRs based on Monte Carlo simulations. The latent growth model approach (red) was unbiased except at the lowest SNR and had lower variance than standard least squares fitting (blue). One fixed “ground truth” GF was created, and different amounts of random noise were added to create four SNR conditions (6, 9, 12, and 15 dB peak SNR). As an example, Panels** a **and** b** show results for a peak SNR of 9 dB. In each panel, the first 15 (of 50) iterations of DPOAE magnitudes are shown by gray lines. To enhance visual clarity, the remaining iterations of DPOAE magnitudes were not plotted. Noise floors are shown by black lines (all 50 iterations plotted). Panel **a**: Light thin pink lines show the predicted GF obtained using the latent growth model approach for each Monte Carlo iteration (all 50 iterations plotted). Thick red line shows the ground truth GF. For comparison, in Panel** b**, light thin blue lines show the corresponding standard least-squares fit for a cubic polynomial (all 50 iterations plotted). See text for fitting details. Thick blue line shows the ground truth GF. Panel **c**: For each iteration, low-level error was computed as the dB difference between the best fitting GF and the true GF at *L*_2_ = 0 dB SPL. Errors for the latent growth model approach are shown by red circles, while errors from the least squares method are shown by blue squares. Individual data points are offset to the left and right of the corresponding SNR for visual clarity. Blue and red lines connect the medians of adjacent distributions to show expected bias in the fits

The excellent fits of predicted GFs to observed data reported in this study are encouraging. However, the participants in this study all had reasonably strong DPOAEs and good SNRs. Fitting the data from participants with poor cochlear function may be more challenging for two potential reasons. First, they are expected to have weaker cochlear amplification, leading to smaller DPOAEs with poorer SNR. Second, it is possible they may have GFs with shapes not readily fit by the latent non-linearity described in this report. To address the first challenge, we used Monte Carlo simulations to quantify the expected accuracy of the latent growth model approach under different SNR conditions. A single underlying GF was created and used as the “ground truth”. On each iteration, random noise was added to the underlying GF in the same manner described in the Interval Estimates section. Different amounts of random noise were added to create four “peak SNR” conditions, where peak SNR was the dB difference between the peak of the GF and the average noise floor value. The noisy GFs were initially fit using the structural equation approach. Fitting accuracy was quantified by computing error as the dB difference between each fit and the “ground truth” GF, evaluated at *L*_2_ = 0 dB SPL. Fifty Monte Carlo iterations were computed for each SNR condition. An example of the fits obtained at 9 dB peak SNR is shown in Fig. 7a. The red circles in Fig. 7c show that the low-level error for the structural equation approach remained small and tightly clustered down to 9 dB peak SNR, with only a slight spreading at 6 dB peak SNR. Standard deviations were 1.87, 1.06, 1.02, and 0.67 dB for 6, 9, 12, and 15 dB peak SNR, respectively. Median errors (−0.71, 0.25, 0.28, −0.03 dB) were near zero, showing that the latent growth model approach remained virtually unbiased even at poor SNRs.

It is also of interest to understand the accuracy of the latent growth model approach compared with “standard” fitting methods that do not include latent structures, nor make use of Rician statistics. Many options for standard fitting exist. For example, polynomial fits could be made on log, semi-log, or linear axes. GF data could be truncated to include only data points with greater than 0, 3, or 6 dB SNR. GF data could be truncated by eliminating portions beyond the GF peak, or the polynomial could be extended to higher orders to include regions above the peak. The coefficients could be found using ordinary or weighted least squares, with the weighting based on SNR or another criterion. We conducted extensive exploratory analyses on this large parameter space. Most options worked well for fitting the portions of the GF truncated to be above the noise floor; however, poor results were obtained for portions of the GF near and below the noise floor, as would be expected from unconstrained extrapolation. When prediction of the GF at low *L*_2_ was the measure of interest, the best results using standard fitting techniques were obtained using the method of least squares to fit a cubic polynomial on linear-linear axes, with the constant term in the polynomial fixed at zero. The fits were made after truncating the GFs between + 3 dB SNR and the GF peak. The fits were subsequently extrapolated down to *L*_2_ = 0 dB SPL. An example of these fits made at 9 dB peak SNR is shown in Fig. 7b. The blue squares in Fig. 7c show that the low-level error for the best standard fitting method had a higher variance than the latent growth model approach, regardless of peak SNR. Standard deviations for the best standard method were 7.22, 3.98, 2.31, and 1.34 dB for 6, 9, 12, and 15 dB peak SNR, respectively. Median errors for the best standard method were substantial (2.74, 2.02, 2.19, 1.17 dB), approaching a 3 dB positive bias at the lowest peak SNRs.

In summary, the approach using a latent growth model with Rician statistics was more robust than standard least squares methods when SNR was poor, performing reasonably well at peak SNRs as low as 6 dB. Simulations and exploratory analyses suggested that some of the superior performance was due to using Rician statistics, and some of it was due to using the latent growth structure. The simulation results shown in Fig. 7 should be interpreted as the most accurate results that can be expected in an ideal testing situation, with recordings from real human and animal participants expected to show higher variability. Nevertheless, these results are encouraging and suggest that the latent growth model approach may be successfully applied to the GFs of participants with mild-to-moderate cochlear hearing loss.

**Fig. 8:**
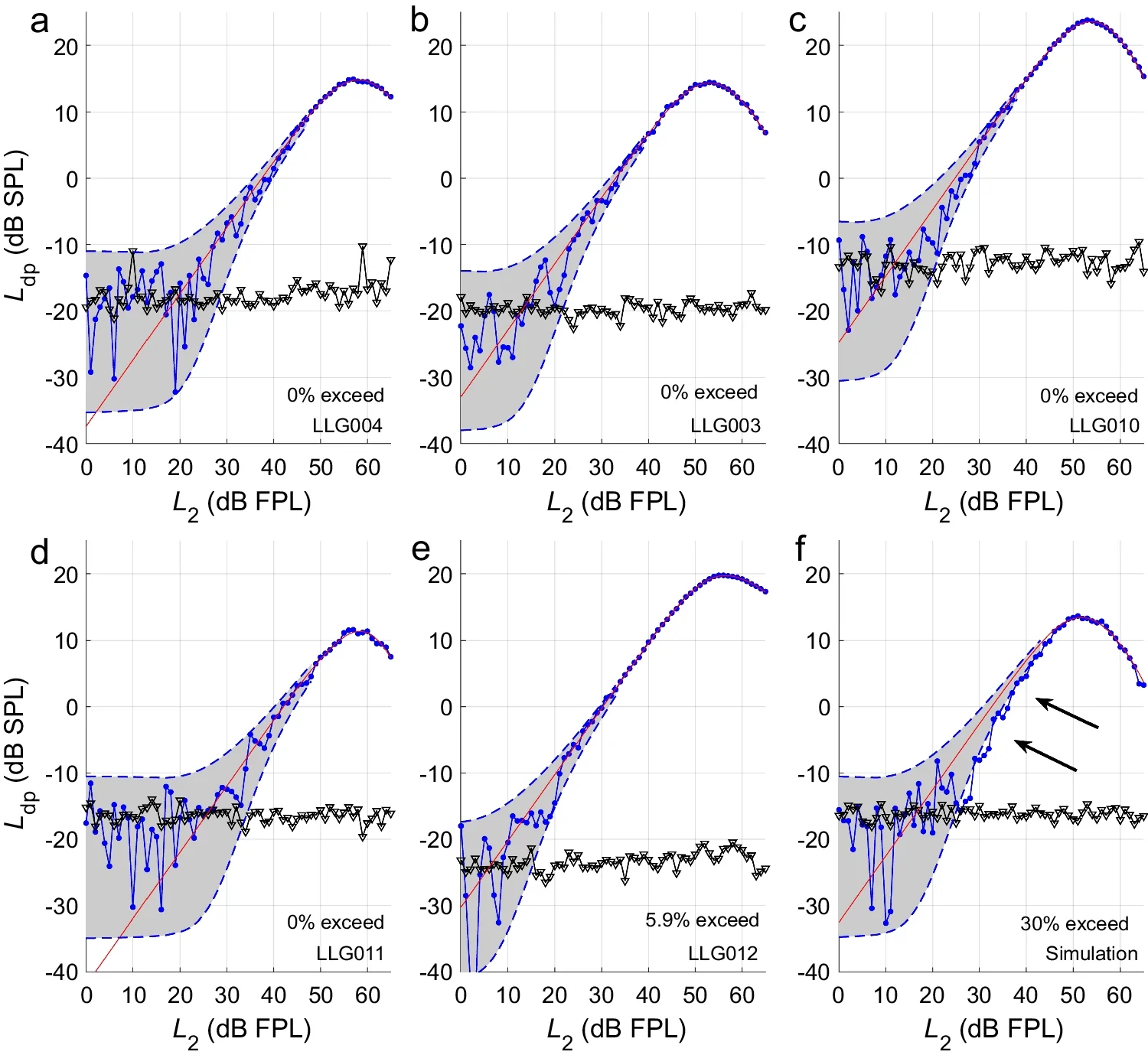
95% tolerance intervals (gray-shaded areas) identifies poor fits between the predicted GF (solid-red curves) and observed GF (blue lines connecting blue dots). In each panel, gray-shaded areas inside dashed-blue lines indicate the 95% tolerance region around the predicted GF. Panels **a–e** show example GFs from participants in this study. Panel **f** shows a simulated GF manipulated to grow most steeply at moderate *L*_2_. This resulted in a poor fit. A simulation was used because all the data in this study were well fit by the structural-equation approach. The poor fit is indicated by a large number of data points (> > 5%) falling outside of the 95% tolerance region. The problematic region is indicated by black arrows

As noted at the beginning of this section, it is possible that fitting the GF data of participants with compromised cochlear function using the structural equation approach may be challenging if GF shapes are different in some way that cannot be readily accounted for by the logistic non-linearity described in this report. For example, the data in Fig. 5 suggest that the steepest slope occurs as *L*_2_ approaches zero, but it is possible that this might not always be the case in some pathological ears. An important question related to this issue is how to know whether the latent growth model approach has provided a good fit for any individual case. In regions where SNR is high, the answer is obvious; however, in regions where SNR is poor, increasing variability makes visual assessment more difficult. One approach would be to verify individual levels using long averaging times to drive the noise floor to very low levels. This might be done for a few select cases (as was done in this study) but this is generally impractical. A more efficient approach is to define a 95%-tolerance region around the predicted GF to show the largest deviations of observed GF data points from the predicted GF that can reasonably be expected. If more than ~ 5% of the data points fall outside the region, this indicates that further careful testing is needed. Examples of this approach are shown in Fig. 8. Tolerance regions were constructed using Monte Carlo simulations (10,000 iterations) to determine expected variation as a function of SNR. Figure 8a–e shows tolerance regions applied to five representative participants. Figure 8f shows a simulation in which the GF was manipulated so that the steepest growth occurred at higher stimulus levels, resulting in a poor fit. Such growth cannot be well fit by the logistic non-linearity used in this study, which always produces the steepest growth as ${|p}_{2}|$ approaches 0 Pa. These examples demonstrate use of the tolerance region to separate variability due to random noise from systematic deviations (compare Fig. 8c and f).

## Discussion

In this paper, we describe a latent growth model (part of the structural equation model framework) to characterize the non-linearity that produced an observed DPOAE GF evoked by the fixed *L*_1_ stimulus paradigm. We used a modified generalized logistic function to model the non-linearity underlying DPOAE generation and the Rician distribution to model the changing GF noise distribution as a function of SNR (Fig. 2). We showed results obtained from a group of young human listeners demonstrating that the described methods accurately predict DPOAE magnitudes at low *L*_2_ stimulus levels (Fig. 6). Using simulations, we demonstrated that this fitting method is robust to GFs with poor SNR (Fig. 7). Finally, we proposed using tolerance regions to identify pathological ears in which GFs may not grow in ways assumed by the underlying latent growth model (Fig. 8).

The latent growth model approach described in this paper may hold at least two significant advantages over standard fitting methods. First, the latent growth model performed well in simulated poor overall SNR conditions (Fig. 7). This was likely due to constraints in the shape of the fit imposed by the use of the modified generalized logistic function and the use of only three free parameters. GFs evoked by the fixed *L*_1_ paradigm exhibit compressive growth to a peak, followed by a rollover (Fig. 1). While some other mathematical models of nonlinear growth (e.g., high-order polynomials (6th–8th order), and Gaussian mixtures) could accurately fit GFs using standard fitting methods and a relatively high number of parameters, they were limited to conditions where GFs had high overall SNRs. In simulated poor SNR conditions, exploratory analyses showed that such models were prone to overfitting and poor performance. In contrast, the latent growth model produced accurate fits in simulations with poor SNR (Fig. 7). While verification of the latent growth model approach is needed using larger data sets that include data from ears with hearing loss, the simulations reported in the current study suggest the latent growth model will perform well on GFs obtained from individuals with mild to moderate cochlear hearing loss.

A second potential advantage of the structural equation approach is obtaining information about the non-linearity underlying GF generation. The form of this non-linearity may yield insights into the state of the cochlea that produced the observed GF. At this initial stage of inquiry, the relationships between the latent non-linearity model and the physiological processes it is meant to represent are unknown. Nevertheless, for the sake of formulating the next hypotheses and guiding future research, some speculation is warranted. The following three paragraphs briefly describe the effect of each latent parameter and an example hypothesis which could be explored in future studies.

The parameter β controls the steepness of growth of the nonlinearity, which is associated with the *L*_2_ at which the peak of the GF occurs (Table 2). Larger values of β (steeper growth of the nonlinearity) are associated with the GF peak occurring at lower *L*_2_. This is consistent with a relationship between β and cochlear gain and suggests the hypothesis that GFs from ears with cochlear hearing loss will be associated with lower β values.

The parameter γ controls the stimulus level at which maximum growth occurs in the nonlinearity, which influences the overall GF peak height and width. This parameter has similar effects on the generalized logistic nonlinearity as an operating point shift in first-order Boltzmann curves: With the operating point at zero, sinusoidal inputs produce output waveforms that are symmetrically distorted in the positive and negative directions, producing maximum odd-order distortion (such as 2*f*_1_-*f*_2_ used in this study). As the operating point shifts away from zero, the magnitude of odd-order distortion decreases in amplitude [24, 47]. This suggests a correlation of γ with GF amplitude; however, γ is correlated with the parameter κ as well, which also has effects on overall level and complicating interpretation. A possible strategy for focusing on the effect of γ is to measure GFs using primary ratios chosen to elicit robust even-order DPOAEs (*f*_2_-*f*_1_) as well as odd-order DPOAEs (2*f*_1_-*f*_2_). Due to the differential effects of operating point on even- and odd-order distortion products, it is hypothesized that ears with larger γ values will have larger *f*_2_-*f*_1_ GFs relative to 2*f*_1_-*f*_2_ GFs, compared with ears having smaller γ values.

The parameter κ has the largest effects on the portions of the GF associated with higher values of *L*_2_ (i.e., the region around the GF peak and second inflection point). This is suggestive of a relationship with spread of excitation along the cochlear partition and two-tone suppression effects. DPs are generated in the region of overlap of cochlear excitation caused by the two primaries, which at low to moderate levels are usually approximated as being near the region of the *f*_2_ cochlear place. In our latent nonlinear model, κ reduced the nominal level (*L*_1_) of the *f*_1_ stimulus seen by the nonlinearity. In other words, the value of κ may describe the *effective L*_1_ at the *f*_2_ cochlear place. This suggests the hypothesis that GFs evoked by primaries with wider frequency ratios will be associated with κ values that result in a larger reduction of *L*_1_, compared to GFs evoked by primaries with narrower frequency ratios.

In addition to further research with human GFs, insights may be obtained by applying the latent growth model to DPOAE GF measurements from animals, along with simultaneous optical coherence tomography (OCT) measurements. Recent OCT measurements have begun to detail the relationships between DPOAEs and intracochlear DPs. This work suggests that similarities in the relationship depend on which specific cochlear structures are under consideration, as well as the stimulus paradigm used [20]. Using growth functions measured from a fixed place along the cochlear partition, Dewey showed compressive growth of the vibration patterns associated with the primary frequencies, as expected [20]. Then using these primary growth functions as inputs, he showed that the resulting DPs could be modeled using a simple Boltzmann function with an operating point shift. For humans, only the ear-canal DPOAEs are available, and the underlying nonlinear vibration pattern must be inferred. It is of interest to understand how the non-linearity obtained by applying the latent growth model relates to the actual vibration of underlying cochlear structures. As preliminary exploration, we used the non-linearities obtained in the current study to infer the growth of the primaries *f*_1_ and *f*_2_ and observed compressive growth patterns, similar to those shown by Dewey in mice ([20], Fig. 2g). While Dewey’s reported results are from a fixed *L*_2_ paradigm, the fixed *L*_1_ paradigm used in our study is expected to give qualitatively similar results in many respects. Additional research is needed to determine the degree of correspondence between the latent non-linearity obtained from structural modeling and actual cochlear vibration patterns. Applying the methods described in the current study to DPOAE and OCT data will address this important question.

In pursuing the structural equation approach to obtain insights into the underlying state of the cochlea, it is important to consider the effects of stimulus levels. In this study, we fixed *L*_1_ at 65 dB FPL and swept *L*_2_ upwards from −2 to 72 dB FPL. This combination was selected as a reasonable choice for obtaining GFs with good SNRs, and for providing proof-of-concept examples for the analysis methods described in this report. These analysis methods are expected to be applicable to other stimulus choices, as well. For example, *L*_1_ could be fixed at lower or higher levels. *L*_2_ could be varied over different ranges, and it could be presented in other orders (e.g., high to low). The latent growth model is a general approach and is applicable to a variety of ways of sampling the (*L*_1_, *L*_2_)-plane, including methods that take a “slice” (e.g., fixed *L*_1_, or fixed *L*_2_, and the “scissors” paradigms), and those that sample more widely to produce “level maps” (e.g., [48, 49]). Regardless of the levels used, a main goal remains to estimate the underlying status and health of cochlear amplification. Since DPOAE amplitude is dependent on stimulus levels [7, 50], it is likely that in its current form, the latent parameter values obtained are at least somewhat dependent on stimulus level choices. We used a modified generalized logistic function as a phenomenological model of latent growth. It is possible that refinements of the model could facilitate representation of cochlear status in a way that is invariant across choices of stimulus level. For example, replacing or supplementing the logistic function with an explicit representation of distributed mechanical to electrical transduction (MET) channels (e.g., [51, 52]) is possible within the latent growth concept. See also the description of Dewey’s work above. Models that consider the effects of middle ear impedance could also be included [53]. These various extended approaches likely confer various advantages and disadvantages, and it remains to be seen which are the most useful for any given problem being considered.

In addition to considering the effects of stimulus levels, the effects of using swept versus discretely presented stimuli should be considered [54]. In this report, we used a swept *L*_2_ presentation and then analyzed the results in discrete, overlapping, 500 ms windows. This resulted in a relatively dense sampling of the GF in steps of 1 dB. Estimating the latent growth parameter values involved finding the smooth curve that best fitted the densely sampled GF. The use of a fitted smooth curve essentially works to “average” across nearby data points, providing additional noise reduction. In measurement paradigms that involve both repeated measurements and fitting smooth curves, noise reduction can occur in two ways: first, by averaging across repeated measurements at a given x-axis value; second, by smoothing across adjacent and nearby values. For a given amount of measurement time, the two types of averaging are sometimes, but not always interchangeable. The extent to which smoothing along the x-axis can be profitable depends on the smoothness of the underlying signal being measured—in other words, the smoothness of the signal in the absence of random noise. For example, if the underlying growth of a signal is linear, then spending all the averaging time at two x-axis values is theoretically no better or worse than spending the same amount of averaging time at ten x-axis values and fitting the ten points with a straight line. As a contrary example, DPOAE fine structure is often “rough”, with many peaks and dips. In this case, relatively dense sampling is required to adequately capture the underlying shapes, and the optimal tradeoff between number of sampling points along the x-axis and the effective amount of averaging time at each point is less clear. The GFs examined in this paper are somewhere in between these two examples. The GFs appear to be quite smooth, but not linear. The minimum number of samples along the x-axis needed to obtain sufficient accuracy is not known. Previous work in our laboratories has successfully applied the model described in this report to GFs with 15 discrete points. Future work may further elucidate this interesting aspect of obtaining and fitting GFs.

### Potential Clinical Utility and Extension to Other Measurements

GFs from the fixed *L*_1_ paradigm may have clinical utility due to their readily interpretable relationship with cochlear health. The methods in this study were used to obtain information about the nonlinearity underlying GF generation, as well as GF growth slopes and inflection points, all of which may be helpful for characterizing cochlear nonlinearity, predicting behavioral audiometric thresholds, estimating loudness perception, and detecting subclinical damage related to aging, noise exposure, and ototoxicity. It has been suggested that different types of cochlear hearing loss, for instance, metabolic versus sensory presbycusis (e.g., damage to the stria vascularis versus hair cells) might result in differing GF slope profiles [3]. A large cohort of participants with differing degrees and types of hearing loss is needed to examine this hypothesis, and the methods described in this report provide a rigorous approach to analysis.

In addressing the issue of differential effects of cochlear pathology on GFs, a potential difficulty is the applicability of the generalized logistic nonlinearity to different types of loss. Based on pilot experiments in our laboratories with humans and animal models, we are cautiously optimistic that the methods in this report can accurately fit GFs associated with any type of cochlear hearing loss. Use of tolerance regions around the predicted GFs may provide a way to identify poor fits (Fig. 8). Should future studies indicate GFs are not well fit in certain circumstances, the specific form of the logistic function could be expanded to include up to six free parameters, thereby increasing flexibility while maintaining the same basic latent structure and preserving the positive features described in this report.

With a focus on methodology, this study was not designed to consider possible differential effects of sex on GFs. Given the small sample and unequal numbers of males and females (9 female, 3 male), it was inappropriate to report differences based on sex. The model was successfully applied to the data from both sexes. It is known that DPOAEs vary as a function of sex [55], and the methods used in this study may help elucidate sex-based differences in GFs in future studies.

Variations of the methods described in this paper can potentially be used to accurately fit other types of GFs. With modifications, the methods might be applied to the growth of DPOAE GFs from the “scissors” paradigm or other variations, such as a fixed *L*_2_ paradigm. These methods may be applied to GFs from stimulus frequency and transient-evoked OAE paradigms. These methods may also be useful for modeling the growth of cochlear microphonics, compound action potentials, and auditory brainstem responses, as well as mechanical motion measurements from the basilar membrane, Reissner's membrane, or organ of Corti. When extending the latent growth model to other measurement types and stimulus paradigms, some caveats should be considered. The fits are dominated by the higher-SNR portions of the GF, so making accurate predictions at stimulus levels near and below the noise floor relies on assumptions and constraints associated with the latent model. It is therefore important to empirically verify the accuracy of low-level growth predictions. The specific latent growth model used for DPOAEs in the fixed *L*_1_ paradigm may not be optimal for other types of GF measurements, and theoretical underpinnings should inform the growth model chosen. Finally, the latent growth model used in this paper was developed for magnitude GFs, and calculations of magnitudes and noise floors were made in the frequency domain. For complex GFs including both magnitude and phase measurements, the distribution would presumably be bivariate normal, and likelihood could be computed directly from that distribution. For phase-only GFs, a circular distribution such as the von Mises distribution would be appropriate.

## Conclusions

We described a latent growth model for characterizing DPOAE GFs. GFs were modeled as a function of primary stimulus levels acting on an underlying nonlinear growth process. A generalized logistic function was used to represent the latent non-linearity, coupled with a generalized linear regression model incorporating the Rician distribution, which is appropriate for fitting GF magnitudes with varying signal-to-noise ratios (SNRs). The parameter values for the non-linearity were fitted to GF data from individual ears, with the output of the model yielding predicted GFs. The method accurately predicted DPOAE magnitudes, including at low stimulus levels where the DPOAEs were below the noise floor.

## Figures and Tables

**Fig. 9:**
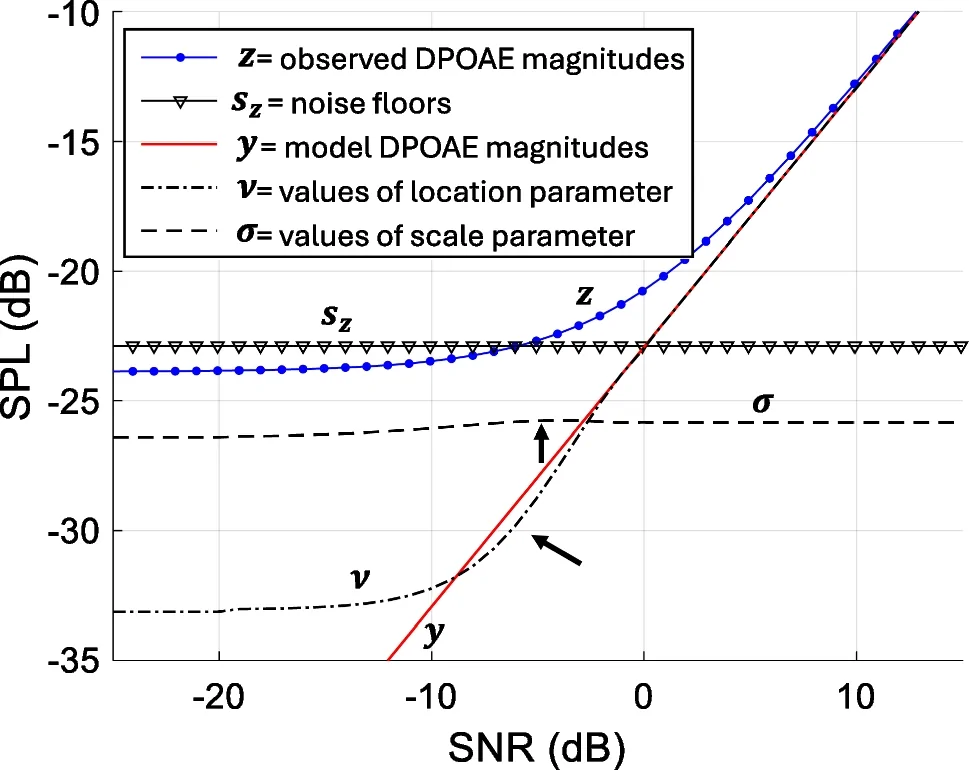
Results of Monte Carlo simulations of DPOAE magnitude and noise floor, along with Rician distribution parameters, as a function of SNR. Values of the simulated noise floor were chosen to yield magnitudes similar to observed human data. For visual clarity, only a restricted range of the SNRs simulated are shown, and data are plotted on a log-log scale. All lines show the mean of 3x10^6^ simulations. The mean simulated DPOAE magnitudes and noise floors (simulated ${\bf{z}}$ and ${{\bf{s}}}_{{\bf{z}}}$) are shown by the blue line with dot markers and black line with triangle markers, respectively. The 1 dB difference between ${\bf{z}}$ and ${{\bf{s}}}_{{\bf{z}}}$ at very low SNRs arises from comparing the mean of ${\bf{z}}$, which is Rayleigh distributed (with a right skew) and the mean of while ${{\bf{s}}}_{{\bf{z}}}$, which is normally distributed. The model DPOAE magnitudes (${\boldsymbol{y}}$), grow linearly in this simulation, as shown by the solid red diagonal line. Parameter values for the location and scale parameters of the Rician distribution (${\boldsymbol{\nu}}$ and ${\boldsymbol{\sigma}}$) are shown by dot-dash and dashed black lines, respectively. See text for explanation of black arrows

## Data Availability

The data sets reported in this study are publicly available and can be obtained from the corresponding author upon reasonable request.

## Appendix

As described in the main text (Methods sub-section entitled “Latent Growth Statistical Model”) and illustrated in the block diagram (Fig. 2), an optimization procedure was used to find the best (in the maximum likelihood sense) predicted GF (${\boldsymbol{y}}$). This was done by iteratively adjusting the nonlinearity parameters which produce the predicted GF. On each iteration, the vectors of Rician distribution parameters ${\boldsymbol{v}}$ and ${\boldsymbol{\sigma}}$ were needed to calculate the likelihood of the proposed predicted GF, given the observed GF data. The values of ${\boldsymbol{\nu}}$ and ${\boldsymbol{\sigma}}$ depend on the noise floor magnitude and the associated SNR of ${\boldsymbol{y}}$. Therefore, for every proposed predicted GF, the elements of ${\boldsymbol{\nu}}$ and ${\boldsymbol{\sigma}}$ were computed by passing the elements of ${\boldsymbol{y}}$ into the link functions g(·) and h(·). This appendix describes the construction and form of the link functions.

In a generalized regression model, such as used in this report, the Rician distribution can function in a similar capacity to the Normal distribution in a standard regression. That is, each observed data point can be thought of as a random draw from a distribution centered at the model fit. Conceptually, for any given observed magnitude with a high SNR, the Rician location parameter ν should be close to the true underlying magnitude. In contrast, at low SNR values, ν should be close to the average noise floor, since the true underlying magnitude is overwhelmed by the noise. The scale parameter of the Rician distribution σ is related to the noise level and should be roughly constant as a function of SNR, since the noise is assumed to be stationary across the GF. These intuitions are mostly correct, with small adjustments needed.

To quantify the relationship between SNR and Rician distribution parameters, Monte Carlo simulations were conducted. Simulation parameters were chosen to yield results similar to human DPOAE GFs. In each iteration, a vector of simulated complex coefficients (as would be obtained from applying Eqs. 1–3) was generated by adding model DPOAE magnitudes (y) to samples of random complex noise. Equations 4–6 were used to obtain the mean signal magnitude $\tt{z}$ and noise floor ${\tt{s}}_{\tt{z}}$. 3 × 10^6^ simulations were conducted at each of 90 SNRs. At each SNR, the Rician distribution was fitted to the simulated magnitudes using MATLAB’s “makedist” function (in the Statistics and Machine Learning Toolbox), which returned maximum likelihood estimates (MLEs) of the parameters ν and σ. Figure 9 shows the mean values that resulted from this process, plotted in dB for clearer visualization.

As expected, the location parameter ν was nearly identical to y when SNR was > 0 dB. However, when SNR values were < -20 dB, ν approached a constant value of ~ 10.26 dB below the noise floor estimate, rather than approaching the noise floor estimate itself. While the transition region between -20 and 0 dB SNR was smooth, ν exhibited values slightly less than y when SNR was between -9 and 0 dB (Fig. 9, black arrow pointing up and to the left). As expected, the scale parameter σ was roughly constant as a function of SNR. However, the value of σ was ∼ 3.52 dB below the noise floor estimate at low SNRs, but ∼ 2.96 dB below the noise floor at high SNRs, with a transition region from -20 to 0 dB SNR (Fig. 9, black arrow pointing upwards).

Because of these observed deviations from simple relationships, the functions linking SNR to Rician parameters were defined piecewise. To avoid confusion when considering signal-to-noise ratio, hereafter in this appendix upper-case letters (SNR) are used to indicate values in dB, while lower-case italicized letters (snr) indicate a ratio. For the location parameter, in the transition region from -20 to 0 dB SNR, a function f was sought such that $\nu=f(snr)\overline{{\tt{s}}_{\tt{z}}}$. The best fitting function was a four-parameter logistic function. For SNR values < -20 dB, the parameter v was equal to a constant times $\overline{{\tt{s}}_{\tt{z}}}$, while at SNR values > 0 dB, the parameter ν was equal to the DPOAE magnitudes (y) (see Eq. A1). In a similar fashion for the scale parameter, in the transition region from -20 to 0 dB SNR, a function f was sought such that $\sigma =f(snr)\overline{{\tt{s}}_{\tt{z}}}$. The best fitting function was a fifth-degree polynomial. For SNR values < 20 dB, the parameter σ was equal to a constant times $\overline{{\tt{s}}_{\tt{z}}}$, while at SNR values > 0 dB, the parameter σ was equal to a second constant times $\overline{{\tt{s}}_{\tt{z}}}$ (see Eq. A2). Note that in Eqs. A1 and A2, the variables $\overline{{\tt{s}}_{\tt{z}}}$ and y are in linear units of mPa, and $snr=y/\overline{{\tt{s}}_{\tt{z}}}$. Values for the coefficients are given in Table A1.

A1 $$\nu=g\;(snr,\;\overline{{\tt{s}}_{\tt{z}}},\;y)=\left\{\begin{array}{cl}0.3069\overline{{\tt{s}}_{\tt{z}}},&if\;SNR\leq-20dB\\\left(\left(d+\left(a-d\right)\right)/\left(1+\left(snr/c\right)^b\right)\right)\overline{s_z},&if\;-20dB<SNR<0dB\\y,&if\;SNR\geq0dB\end{array}\right.$$

A2 $$\sigma=h(snr,\overline{{\tt{s}}_{\tt{z}}})=\left\{\begin{array}{cl}0.667\overline{{\tt{s}}_{\tt{z}}},&if\;SNR\leq-20dB\\\left(p_4{snr}^4+p_3{snr}^3+p_2{snr}^2+p_1snr+p_0\right)\overline{{\tt{s}}_{\tt{z}}},&if-20dB < SNR< 0dB\\0.711\overline{{\tt{s}}_{\tt{z}}},&if\;SNR\geq0dB\end{array}\right.$$

**Table A1:** List of coefficient values for the functions linking SNR to the Rician coefficients ν and σ (Eqs. A1 and A2)

$g(snr,\overline{{\tt{s}}_{\tt{z}}},y)$		$h(snr,\overline{{\tt{s}}_{\tt{z}}})$		
constant	logistic	constant	polynomial	constant
0.3069	a=0.309983	0.667	${p}_{4}= 0.676563$	0.711
	b=3.605490		${p}_{3}=-1.462575$	
	c=0.828416		${p}_{2}= 0.922066$	
	d=1.331526		${p}_{1}=-0.094255$	
			${p}_{0}=$ 0.670568	

## In this report, variables in regular typeface indicate scalar values, while variables in bold typeface indicate vectors or matrices.

${L}_{1}$: Level of the lower frequency primary $({f}_{1}$) in decibels
${L}_{2}$: Level of the higher frequency primary $({f}_{2}$) in decibels
${L}_{dp}$: Level of the $2{f}_{1}-{f}_{2}$ distortion product in decibels
${|p}_{2}|$: Pressure magnitude of the higher frequency primary $({f}_{2}$)
${|p}_{dp}|$: Pressure magnitude of the $2{f}_{1}-{f}_{2}$ distortion product
M: Number of sweeps in recording
n: Number of samples in each analysis frame
N: Number of analysis frames
${\boldsymbol{X}}$: Solution matrix for least-squares fit
${\boldsymbol{a}}$: Recorded ear canal pressures in an analysis frame
${\boldsymbol{b}}$: Coefficients for the least-squares fit
$\widehat{{\boldsymbol{b}}}$: Best estimates of the least-squares fit coefficients
$$\boldsymbol{w}$$: Bisquares weights
${z},{\boldsymbol{z}}$: Least squares fit coefficients expressed in complex form (vector is across sweeps)
$\overline{\it{z}}$: Complex mean across sweeps
$\tt{z},\bf{z}$: Magnitude of the DPOAE GF (vector is across *L*_2_)
${\tt{s}}_{\tt{z}},{\bf{s}}_{\bf{z}}$: Weighted estimator of the standard deviation of the mean (vector is across *L*_2_)
$\overline{{\tt{s}}_{\tt{z}}}$: Mean noise floor
${{\boldsymbol{p}}}_{1}$: Primary stimulus waveform for the lower frequency, *f*_1_
${{\boldsymbol{p}}}_{2}$: Primary stimulus waveform for the lower frequency, *f*_2_
${\boldsymbol{p}}$: Combined stimulus waveform, the sum of the two primaries
${\boldsymbol{q}}$: Output of the compressive nonlinearity
${\boldsymbol{y}}$: Underlying (model) GF as a function of *L*_2_
${\boldsymbol{x}}$: *L*_2_ Stimulus level, expressed in linear units of mPa
$\beta ,\gamma$: Parameters for the latent nonlinearity
κ: Latent parameter for scaling *L*_1_
$\mathrm{S}\mathrm{N}\mathrm{R}$: Signal-to-noise ratio (dB)
snr: Signal-to-noise ratio (linear)
${\boldsymbol{\nu}}$: Location parameter of the Rician distribution (vector is across *L*_2_)
${\boldsymbol{\sigma}}$: Scale parameter of the Rician distribution (vector is across *L*_2_)
g(·): Link function returning the Rician parameter ν
h(·): Link function returning the Rician parameter σ
$f\left(\tt{z};{\nu},{\sigma} \right)$: Probability density function of the Rician distribution
$\mathcal{L}\left({\boldsymbol{\nu}},{\boldsymbol{\sigma}};{\bf{z}}\right)$: Likelihood function for the Rician distribution
$\left\{\widehat{\beta },\widehat{\gamma },\widehat{\kappa }\right\}$: Maximum likelihood estimates for the nonlinearity

## Footnotes

1 Since the weighting vector ${\boldsymbol{w}}$ sums to one, ${\sigma }^{2}=\sum {\boldsymbol{w}}\left(\left({\boldsymbol{z}}-\overline{{z} }\right){\left({\boldsymbol{z}}-\overline{{z} }\right)}^{\boldsymbol{*}}\right)$ is a biased estimator of the variance because one degree of freedom was used to calculate $\overline{{z}}$. In the unweighted version of the variance calculation, $\sum \left(\left({\boldsymbol{z}}-\overline{{z} }\right){\left({\boldsymbol{z}}-\overline{{z} }\right)}^{\boldsymbol{*}}\right)$ should be divided by M-1 rather than M. Here, the weights sum to one, effectively dividing by M, which makes the estimate of the variance slightly smaller than it should be. This is corrected by dividing by $1-{M}^{-1}$, where M is the number of sweeps. This works because the sum of the weights is 1, and ${M}^{-1}$ is 1 divided by the number of sweeps, or in other words, the average value of a single weighting element of ${\boldsymbol{w}}$. The ${M}^{-1}$ in the numerator of Eq. 6 converts the variance to the standard error of the mean by dividing by the number of elements in ${\boldsymbol{z}}$. In summary, the denominator in front of the summation sign adjusts for the degree of freedom already used to calculate the mean, and the numerator converts variance to the standard error.

## Code Availability

The analysis codes used in this study are available on the corresponding author’s GitHub repository at https://github.com/myKungFu/DPOAE-GF.

## Declarations

### Use of AI

No traditional or generative AI technologies were used for any aspect of this project.
