# Correction: A Structural Equation Approach to Characterizing Growth and Nonlinearity Underlying Distortion Product Otoacoustic Emissions

**DOI:** 10.1007/s10162-026-01064-w

**Published:** 2026-06-22

**Authors:** Shawn S. Goodman, M. Ehsan Khalili, Julia H. Roemen, Ishan Bhatt, Skyler G. Jennings, Jeffery T. Lichtenhan, Sumitrajit Dhar

**Affiliations:** 1 Department of Communication Sciences and Disorders, University of Iowa, Iowa City, IA USA; 2 Department of Communication Sciences and Disorders, University of Utah, Salt Lake City, UT USA; 3 Dept. of Otolaryngology, University of South Florida, Tampa, FL USA; 4 Turner Scientific, Jacksonville, IL USA; 5 Roxelyn and Richard Pepper Dept. of Communication Sciences and Disorders, Northwestern University, Evanston, IL USA; 6 Knowles Hearing Center, Northwestern University, Evanston, IL USA

**Correction: Journal of the Association for Research in Otolaryngology**

10.1007/s10162-026-01057-9

The original online version of this article was revised to correct the presentation of Greek characters in Equation 9. The corrected equation is as follows:

9 $$f\left(z;\;v,\sigma\right)=\frac z{\sigma^2}e^{\left(-\left(z^2+v^2\right)/2\sigma^2\right)}I_0\left(\frac{zv}{\sigma^2}\right),$$

The original article has been corrected.

